# Engineered CAR‐NKT Extracellular Vesicles Suppress Tumor Progression and Enhance Antitumor Immunity

**DOI:** 10.1002/advs.202521623

**Published:** 2026-01-20

**Authors:** Xiaopei Hao, Chengming Qu, Yanzhao Zhou, Xiaoqian Wang, Xiangjun Qian, Xun Chen, Feng Han, Xiaokai Zhang, Yiyi Ji, Han Li, ChengWei Ju, Peng Xia, Weiwei Tang, Hao Zhuang, Jinxue Zhou

**Affiliations:** ^1^ Department of Hepatobiliary and Pancreatic Surgery The Affiliated Cancer Hospital of Zhengzhou University & Henan Cancer Hospital Zhengzhou 450008 China; ^2^ Hepatobiliary Center, The First Affiliated Hospital of Nanjing Medical University, Key Laboratory of Liver Transplantation Chinese Academy of Medical Sciences, NHC Key laboratory of Hepatobiliary Cancers Nanjing 210029 China; ^3^ Department of Chemistry, Department of Biochemistry and Molecular Biology The University of Chicago Chicago IL 60637 USA; ^4^ Zhongnan Hospital of Wuhan University, TaiKang Center for Life and Medical Sciences, Clinical Medicine Research Center for Minimally Invasive Procedure of Hepatobiliary & Pancreatic Diseases of Hubei Province Wuhan University Wuhan 430071 China; ^5^ Department of Medical Oncology The Affiliated Cancer Hospital of Zhengzhou University & Henan Cancer Hospital Zhengzhou 450008 China; ^6^ Department of Biomedical Engineering Duke University Durham North Carolina 27708 USA

**Keywords:** cancer therapy, CAR‐NKT, extracellular vesicles, immunotherapy, TM4SF1 nanobody

## Abstract

Chimeric antigen receptor‐engineered natural killer T (CAR‐NKT) cell therapy represents a promising and innovative strategy in cancer immunotherapy, but is limited by acute toxicity and adverse effects, restricting broader clinical application despite durable responses. In this study, a novel nanobody targeting TM4SF1 is developed, which replaced the conventional single‐chain variable fragment (scFv) in the design of CAR^TM4SF1^‐NKT cells. Moreover, CAR^TM4SF1^‐extracellular vesicles (EVs) therapy as an optimized alternative to direct CAR‐NKT cell administration is introduced. Compared with conventional CAR^TM4SF1^ engineered cells, CAR^TM4SF1^‐EVs demonstrated superior antitumor efficacy while significantly reducing toxicity. This findings revealed that CAR^TM4SF1^‐EVs selectively targeted TM4SF1‐expressing tumor cells in both in vitro and in vivo models. In hepatocellular carcinoma (HCC) mouse models, CAR^TM4SF1^‐EVs induced immunogenic cell death (ICD) and effectively suppressed tumor growth and metastasis. The therapeutic efficacy of CAR^TM4SF1^‐EVs is primarily attributed to their ability to remodel the immunosuppressive tumor microenvironment (TME), notably by enhancing CD8⁺ T cell activity and eliciting robust antitumor immune responses. Furthermore, CAR^TM4SF1^‐EVs synergized with Immune Checkpoint Blockade (ICB) therapy, leading to durable antitumor immune memory. Collectively, these findings establish CAR^TM4SF1^‐EVs therapy as a safe and effective strategy for targeted cancer immunotherapy, underscoring its potential for clinical application.

## Graphical abstract

TM4SF1‐nanobody engineered CAR‐natural killer T–derived extracellular vesicles (CAR^TM4SF1^‐EVs) provide a cell‐free alternative to CAR‐NKT therapy, achieving potent, targeted antitumor activity with reduced toxicity. CAR^TM4SF1^‐EVs induce immunogenic cell death, remodel the tumor microenvironment, and enhance CD8⁺ T‐cell antitumor immunity. They further synergize with immune checkpoint blockade, providing a safe and effective strategy for cancer immunotherapy.

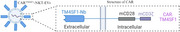

## 1 Introduction

Recent advancements in chimeric antigen receptor (CAR) engineering have significantly propelled the field of cancer immunotherapy, particularly in the treatment of hematologic malignancies, where CAR‐T cell therapies have demonstrated unprecedented efficacy, suggesting a novel direction for cancer treatment.^[^ ^1^, ^2^ ^]^ Despite these remarkable outcomes, CAR‐T cell therapies are hindered by several limiting factors, including severe toxicities such as cytokine release syndrome (CRS), serious off‐target effects in solid tumors, and CAR‐T cell — related encephalopathy syndrome (CRES), which substantially restrict their clinical applicability.^[^ ^3^, ^4^ ^]^ Moreover, challenges such as antigen escape, limited persistence of CAR‐T cells, and the immunosuppressive tumor microenvironment markedly contribute to therapeutic resistance and relapse.^[^ ^5^ ^]^

Natural killer T (NKT) cells have emerged as a promising focus in cancer immunotherapy because of their unique tumor‐targeting capabilities and reduced risk of graft‐versus‐host disease (GvHD).^[^ ^6^ ^]^ Studies have demonstrated that chimeric antigen receptor natural killer T (CAR‐NKT) cells can efficiently eliminate CD1d‐expressing M2‐type tumor‐associated macrophages and significantly promote epitope spreading, thereby enhancing endogenous T‐cell responses against tumor‐associated neoantigens.^[^ ^7^, ^8^, ^9^ ^]^ However, the rarity of NKT cells and the susceptibility of allogeneic cell products to host immune rejection remain significant barriers to their widespread application, necessitating further research and technological innovations to address these limitations.^[^ ^10^ ^]^

To address these challenges, we focused on developing extracellular vesicle (EV)‐based therapies engineered with CARs. CAR‐EVs, nanoscale particles secreted by CAR‐engineered cells, offer several distinct advantages. First, their small size (≈100–200 nm) allows them to effectively penetrate solid tumors, overcoming the physical barriers posed by the tumor microenvironment.^[^ ^11^, ^12^, ^13^, ^14^, ^15^ ^]^ Second, compared with traditional CAR‐based cell therapies, CAR‐EVs significantly reduce cytokine release‐associated toxicity while retaining high specificity and potent cytotoxicity against tumor cells.^[^ ^16^ ^]^ Furthermore, as they lack viable cellular components, CAR‐EVs do not trigger graft‐versus‐host reactions, thereby demonstrating enhanced safety and broader applicability in allogeneic settings.^[^ ^17^, ^18^ ^]^ These advantages position CAR‐EVs as promising candidates for CAR‐based cell therapy with broad clinical potential and applicability.

TM4SF1, a small plasma membrane glycoprotein characterized by its tetraspanin architecture, exhibits markedly elevated expression on the neoplastic cell surfaces across 16 distinct malignancies—including HCC—while remaining virtually undetectable in adjacent non‐tumorous tissues.^[^ ^19^ ^]^ Notably, its expression in normal vasculature is restricted to faint endothelial signals. This striking tumor‐selective expression profile positions TM4SF1 as a highly promising therapeutic target in oncology.^[^ ^20^, ^21^ ^]^ Consequently, we selected TM4SF1 as the target for our CAR design and then developed an innovative TM4SF1‐specific nanobody. Compared with single‐chain variable fragment (scFv) antibodies, nanobodies are smaller in size and thereby offer notable advantages, such as superior tumor penetration.^[^ ^17^, ^22^ ^]^ Furthermore, their flexible structural design not only meets the engineering requirements of CARs but also aligns well with the application of EV‐based therapies.^[^ ^13^, ^17^ ^]^ It has also been reported that nanobodies provide superior targeting specificity and stability, significantly increasing the overall efficacy of CAR‐based therapies.^[^ ^23^, ^24^ ^]^ This advancement opens new avenues for cancer immunotherapy, demonstrating the transformative potential of nanobody‐based approaches in tumor treatment.

**Scheme 1:**
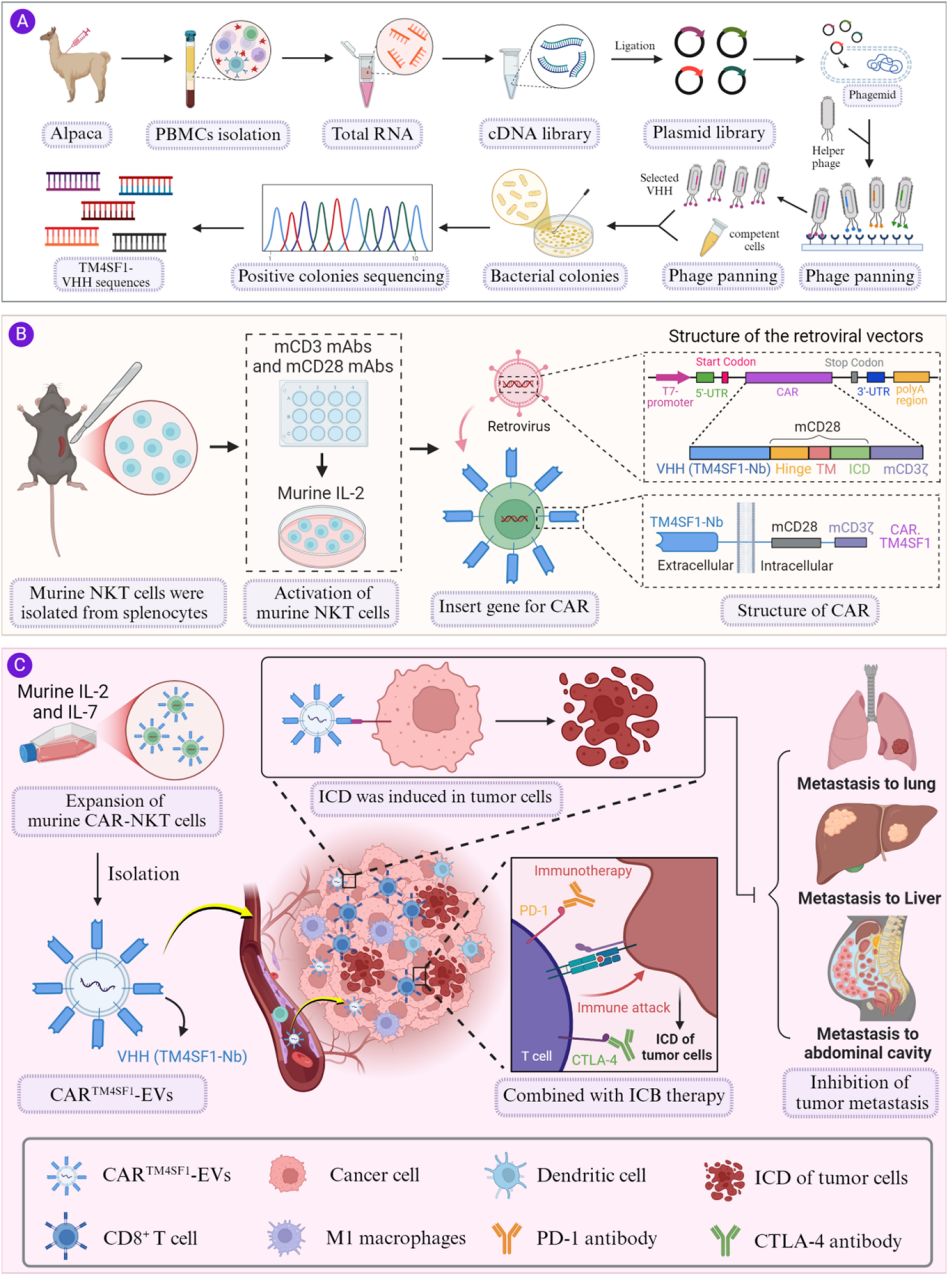
Schematic diagram of TM4SF1 nanobody‐based CAR‐NKT‐derived EVs that induce ICD in HCC and enhance the efficacy of immune checkpoint blockade. Created with BioRender.com. Publication rights have been obtained.

Building upon these findings, we engineered a novel TM4SF1‐specific nanobody into the extracellular domain of CAR NKT cells and isolated their EVs, thereby generating CAR^4SF1^‐EVs. Our findings revealed that CAR^4SF1^‐EVs effectively targeted and eliminated tumor cells while alleviating the immunosuppressive tumor microenvironment, thereby activating robust antitumor immune responses. When combined with programmed death protein‐1 (PD‐1) antibodies or cytotoxic T lymphocyte‐associated protein 4 (CTLA‐4), CAR^4SF1^‐EVs further increased antitumor efficacy and successfully induced immune memory, resulting in sustained tumor suppression (**Scheme** **1**). This study offers a novel perspective on the application of CAR‐EVs in the treatment of solid tumors and underscores their immense potential as an optimized next‐generation CAR therapy strategy.

## 2 Results

### 2.1 High TM4SF1 Expression is Closely Associated with HCC Progression and Poor Prognosis

TM4SF1 has been recognized as a tumor‐selective surface glycoprotein predominantly expressed in a variety of epithelial malignancies, including HCC, while remaining scarcely detectable in normal parenchymal tissues.^[^ ^19^, ^25^ ^]^ This tumor‐restricted distribution underscores its potential as a promising therapeutic target in solid tumors.^[^ ^19^, ^26^, ^27^ ^]^ Building on these insights, we next analyzed TM4SF1 expression patterns and their clinical significance in HCC to further validate its relevance for CAR‐based therapeutic development.

In the initial phase of this study, bioinformatics analysis of data obtained from The Cancer Genome Atlas (TCGA) database and the Genotype‐Tissue Expression (GTEx) database revealed significantly higher TM4SF1 expression in human HCC tissues than in normal liver tissues (Figure S1A, Supporting Information). We subsequently investigated the relationships between TM4SF1 expression and overall survival (OS) and disease‐free survival (DFS) in HCC patients. The results indicated that high TM4SF1 expression was significantly correlated with poor prognosis (Figure S1B,C, Supporting Information). A previous study analyzing RNA from 50 paired HCC and adjacent normal liver tissues reported that TM4SF1 was upregulated in 80% of HCC samples.^[^ ^28^ ^]^ To further confirm TM4SF1 expression in HCC tissues, we collected 36 paired HCC and adjacent normal liver tissue samples for quantitative real‐time polymerase chain reaction (qRT‐PCR) analysis (Figure S1D, Supporting Information), which consistently showed significantly elevated TM4SF1 expression in tumor tissues.

Collectively, these results confirm that TM4SF1 is markedly overexpressed in HCC and is closely associated with aggressive tumor progression and poor clinical outcomes, underscoring its potential as a biologically and clinically significant therapeutic target in HCC.

### 2.2 Screening and Purification of the TM4SF1 Nanobody

**Figure 1:**
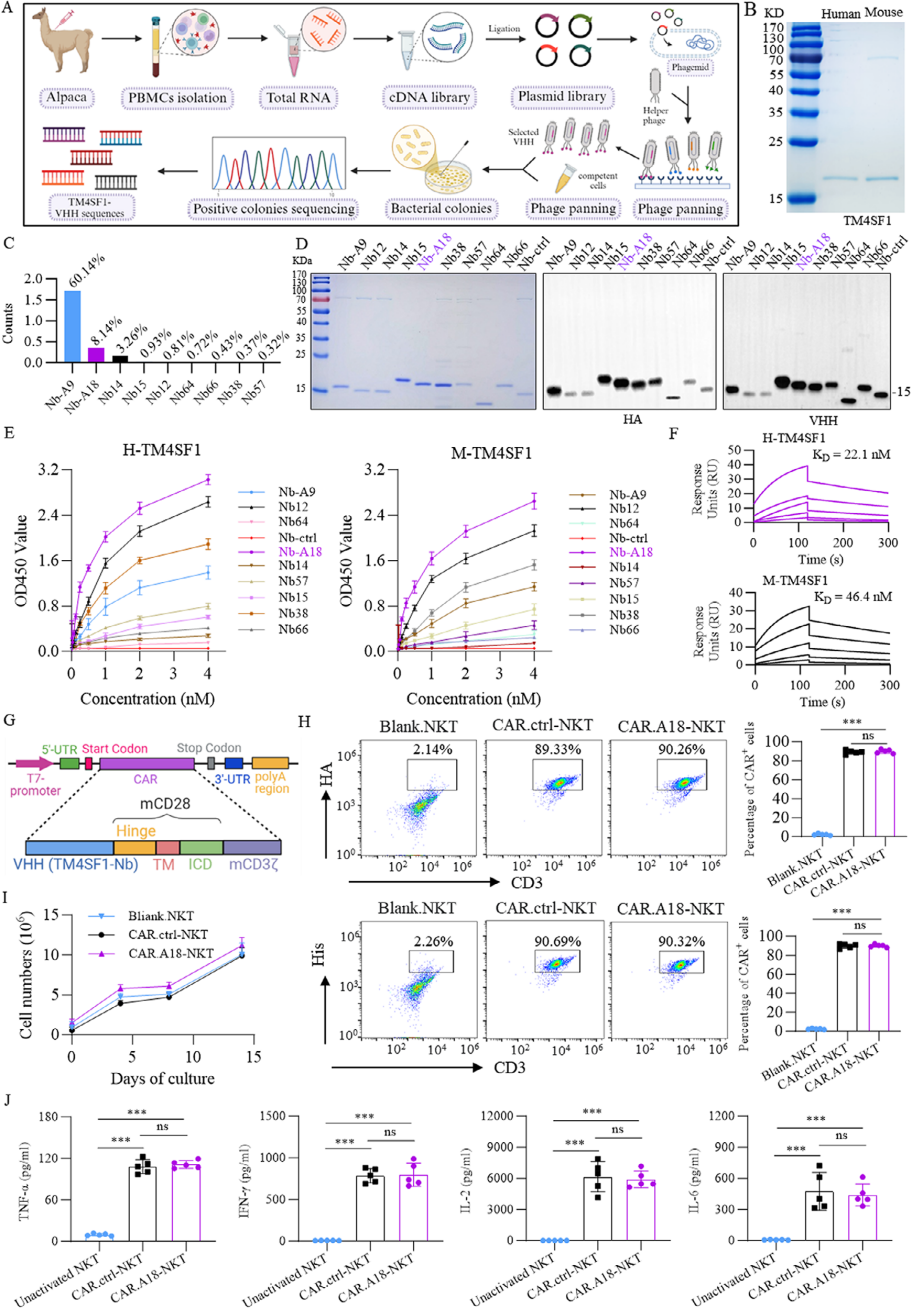
Construction and Characterization of CAR.A18‐NKT Cells. A) Schematic for screening TM4SF1‐specific nanobodies using phage display technology. B) SDS‒PAGE gel showing the purified human and murine TM4SF1 proteins. C) Sequencing results displaying the frequency of potential positive nanobody sequences. D) Identification of 10 screened and purified TM4SF1 nanobodies. E) ELISA results showing the binding activity of TM4SF1 nanobodies with human and murine TM4SF1 proteins (*n* = 3). F) SPR analysis of the binding activity of the TM4SF1‐A18 nanobody with the human and murine TM4SF1 proteins. G) Schematic of the retroviral vectors encoding CAR.TM4SF1 and CAR.Ctrl. H) Representative flow cytometry plots showing the proportions of constructed CAR‐NKT cells. I) In vitro expansion of CAR‐NKT cells *(n* = 3). J) TNF‐α, IFN‐γ, IL‐2, and IL‐6 levels in the supernatants of CAR‐NKT cell cultures measured via ELISA (*n* = 5). Quantitative data are presented as mean ± standard deviation (SD). Statistical significance was calculated using one‐way ANOVA with Tukey's post‐test (H, J) or two‐way ANOVA with Tukey's post‐test (I). Statistical significance is indicated as ns (not significant), *p < 0.05, **p < 0.01, ***p < 0.001.

Previous studies demonstrated that the use of TM4SF1‐specific antibodies, antibody‒drug conjugates (ADCs), and third‐generation chimeric antigen receptor T (CAR‐T) cells achieved significant antitumor effects in cancer types with high TM4SF1 expression, such as lung, liver, and ovarian cancers.^[^ ^29^, ^30^, ^31^, ^32^ ^]^ However, these studies utilized primarily full‐length antibodies or scFvs. In contrast, nanobodies, with their small molecular weight (≈15 kDa), offer superior tumor penetration, increased stability, and decreased immunogenicity.^[^ ^22^ ^]^ Furthermore, the nanobody structure lacks an Fc domain, enabling nanobodies to be efficiently produced using prokaryotic expression systems and making them highly promising tools for biomedical research and clinical applications.^[^ ^13^ ^]^ On the basis of these advantages, we developed a TM4SF1‐specific nanobody following a flowchart (**Figure** **1**A). First, we purified human and murine TM4SF1 proteins (≈22 kDa; Figure 1B). Using the human TM4SF1 protein as the antigen, we performed three rounds of biopanning with an alpaca nanobody phage display library. This process yielded nine highly enriched nanobody sequences, with their frequency distributions shown in Figure 1C.

We subsequently successfully expressed and purified the nine TM4SF1 nanobodies and a control nanobody using an *Escherichia coli* expression system. SDS‒PAGE and HA‐tag and HIS‐tag antibody validation confirmed the high purity of the purified TM4SF1 nanobodies (Figure 1D). To evaluate their binding affinities, we conducted enzyme‐linked immunosorbent assays (ELISA) with human and murine TM4SF1 proteins. Our results revealed that the A18 nanobody exhibited the highest affinity for both the human and murine TM4SF1 proteins compared with the control and other nanobodies (Figure 1E). We subsequently performed surface plasmon resonance (SPR) to validate the binding kinetics of the A18 nanobody. The results revealed dissociation constants (K_D_) of 22.1 and 46.4 nM for the human and murine TM4SF1 proteins, respectively (Figure 1F). In conclusion, we successfully screened and identified a TM4SF1‐specific nanobody with high affinity for both human and murine TM4SF1 proteins. Among the candidates, the A18 nanobody, which presented the strongest affinity, was selected for subsequent functional studies.

### 2.3 Construction and Characterization of CAR^TM4SF1^ Cells

Previous studies have demonstrated that autologous CAR‐NKT cells exhibit superior antitumor activity to CAR‐T cells in various syngeneic tumor models.^[^ ^10^ ^]^ To evaluate the therapeutic efficacy and mechanisms of CAR‐NKT cells, we developed a method to construct CAR‐NKT cells that target TM4SF1 using murine NKT cells. Briefly, NKT cells were isolated from the spleens of C57BL/6J mice and stimulated on plates coated with 1 mg mL^−1^ anti‐mCD3 and 1 mg mL^−1^ anti‐mCD28 monoclonal antibodies (mAbs). The cells were activated in complete RPMI 1640 with murine IL‐2 (30 U mL^−1^) for 48 h. NKT cells were then transduced with retroviral vectors encoding CAR^4SF1^ or CAR^ctrl^, which contained the sequence for Nb‐A18 or Nb‐ctrl, respectively (Figure 1G). To confirm the successful generation of CAR^4SF1^ (CAR. A18‐NKT) cells, flow cytometry was used to analyze the expression of CD3 and HA‐tag or His‐tag on NKT cells. Flow cytometry revealed that the percentages of CD3^+^ HA^+^ and CD3^+^ His^+^ cells among CAR.A18‐NKT cells and CAR.ctrl‐NKT cells were significantly greater than those among unmodified NKT (Blank.NKT) cells (Figure 1H). Additionally, both CAR.A18‐NKT and CAR.ctrl‐NKT cells exhibited comparable proliferation rates to Blank.NKT cells during in vitro expansion (Figure 1I). Moreover, compared to unactivated NKT cells (unstimulated with mCD3, mCD28, or IL‐2), CAR.A18‐NKT and CAR.ctrl‐NKT cells produced high levels of TNF‐α, IFN‐γ, IL‐2, and IL‐6 (Figure 1J). In summary, we successfully constructed CAR.A18‐NKT cells, which demonstrated robust proliferation and cytokine production capabilities in vitro. These findings provide a solid foundation for further functional and therapeutic evaluations of CAR‐NKT cells targeting TM4SF1.

### 2.4 Purification and Characterization of CAR^TM4SF1^‐EVs

**Figure 2:**
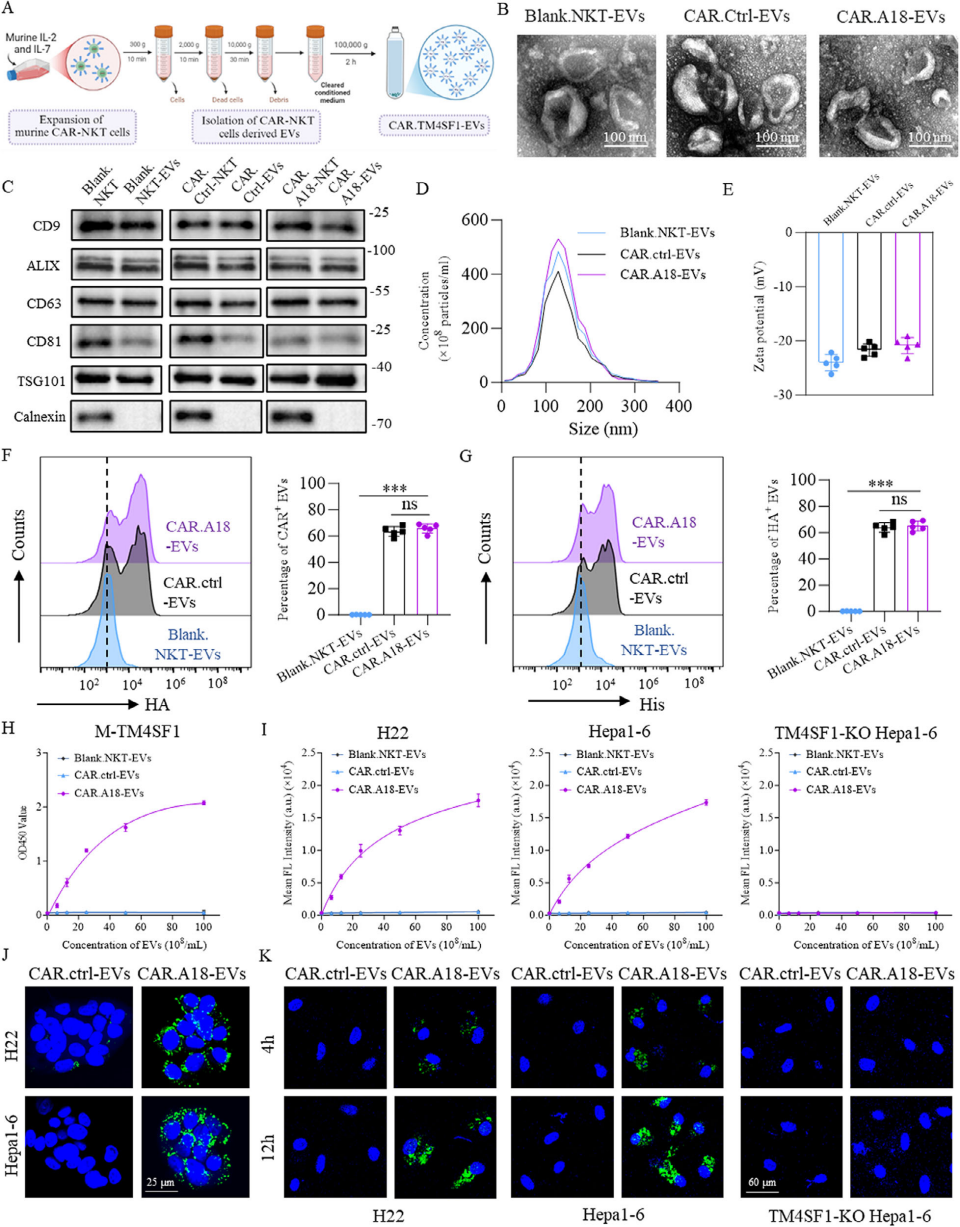
Purification and Characterization of EVs Released by CAR‐NKT Cells. A) Schematic for constructing CAR‐EVs using CAR.A18‐NKT cells. B) Representative TEM images of Blank.NKT‐EVs, CAR.ctrl‐EVs, and CAR.A18‐EVs. Scale bar, 100 nm. C) Western blot analysis of EV biomarkers, including CD9, ALIX, CD63, CD81, and TSG101, in Blank.NKT‐EVs, CAR.ctrl‐EVs, and CAR.A18‐EVs. D) Size distribution of Blank.NKT‐EVs, CAR.ctrl‐EVs, and CAR.A18‐EVs measured by NTA. E) Zeta potential measurements of Blank.NKT‐EVs, CAR.ctrl‐EVs, and CAR.A18‐EVs (*n* = 5). F) Flow cytometry analysis of the number of Blank.NKT‐EVs, CAR.ctrl‐EVs, and CAR.A18‐EVs expressing the HA tag (*n* = 5). G) Flow cytometry analysis of the number of Blank.NKT‐EVs, CAR.ctrl‐EVs, and CAR.A18‐EVs expressing VHH proteins (*n* = 5). H) ELISA results showing the binding affinity of Blank.NKT‐EVs, CAR.ctrl‐EVs, and CAR.A18‐EVs to murine TM4SF1 (*n* = 3). I) Cell‐based ELISA demonstrating the binding of Blank.NKT‐EVs, CAR.ctrl‐EVs, and CAR.A18‐EVs to H22, Hepa1‐6, and TM4SF1‐KO Hepa1‐6 cells (*n* = 3). J) IF staining showing the binding of CAR.ctrl‐EVs (green fluorescence) and CAR.A18‐EVs (green fluorescence) to the membranes of H22 and Hepa1‐6 cells. Scale bar, 25 nm. K) IF analysis of the internalization of CAR.ctrl‐EVs (green fluorescence) and CAR.A18‐EVs (green fluorescence) by H22, Hepa1‐6, and TM4SF1‐KO Hepa1‐6 cells. Scale bar, 60 nm. Quantitative data are presented as mean ± SD. Statistical significance was calculated using one‐way ANOVA with Tukey's post‐test (E, F, G) or two‐way ANOVA with Tukey's post‐test (H, I). Statistical significance is indicated as ns (not significant), *p < 0.05, **p < 0.01, ***p < 0.001.

While CAR‐T‐cell‐ and CAR‐NKT‐cell‐based immunotherapies have made significant progress in both clinical and preclinical studies for hematological and solid tumors, their application is often hindered by severe side effects, such as CRS and multiorgan toxicity.^[^ ^10^, ^33^ ^]^ EVs, as natural products of cells, exhibit high stability and prolong the circulation half‐life in the bloodstream.^[^ ^34^ ^]^ Previous reports have demonstrated that cytotoxic T lymphocyte (CTL)‐derived EVs, which contain surface membrane molecules such as CD3, CD8, and TCR, ensure targeted tumor cell killing.^[^ ^18^, ^35^ ^]^ Given their low immunogenicity and reduced toxicity, EVs derived from CAR.A18‐NKT cells may offer direct, specific cytotoxicity against TM4SF1‐overexpressing tumors while avoiding severe adverse effects. To explore this potential, we isolated and purified CAR.A18‐EVs from CAR^4SF1^‐NKT cells. Different types of NKT cells were extensively expanded in vitro using IL‐2 and IL‐7, and their culture supernatants were collected for EV isolation and purification via ultracentrifugation (**Figure** **2**A). Transmission electron microscopy (TEM) revealed that Blank.NKT‐EVs, CAR.A18‐EVs, and CAR.ctrl‐EVs displayed characteristic “cup‐shaped” or “disc‐shaped” morphologies (Figure 2B). Furthermore, we analyzed classical EV biomarkers (CD9, ALIX, CD63, CD81, and TSG101), all of which were positively expressed, while the negative marker calnexin was absent (Figure 2C). Nanoparticle tracking analysis (NTA) revealed that all three types of EVs presented average particle sizes ranging from 100 nm to 200 nm, which was consistent with the observations from TEM (Figure 2D). Additionally, all EVs exhibited a negative zeta potential, suggesting enhanced stability and dispersion under physiological conditions (Figure 2E). Importantly, flow cytometry confirmed the robust expression of CAR proteins (HA‐tag and His‐tag) on the surface of CAR.A18‐EVs and CAR.ctrl‐EVs compared with Blank.NKT‐EVs (Figure 2F,G). These results demonstrated the successful construction of CAR.A18‐EVs, with abundant TM4SF1 nanobody expression on their surface.

To assess the specificity of CAR.A18‐EVs for human and murine TM4SF1 proteins, ELISA and immunofluorescence (IF) assays were performed. Our results showed that CAR.A18‐EVs displayed significantly greater binding affinity for human and murine TM4SF1 proteins than Blank.NKT‐EVs and CAR.ctrl‐EVs (Figure 2H; Figure S2, Supporting Information). The cell‐based ELISA results further revealed strong binding of the CAR.A18‐EVs to TM4SF1‐high H22 and Hepa1‐6 cells, whereas no binding was observed with TM4SF1‐knockout Hepa1‐6 cells (Figure 2I). IF assay corroborated these findings, indicating that CAR.A18‐EVs (green fluorescence) bound extensively to the surface of H22 and Hepa1‐6 cells but not to TM4SF1‐knockout Hepa1‐6 cells (Figure 2J). Furthermore, when cocultured with H22 or Hepa1‐6 cells, CAR.A18‐EVs demonstrated time‐dependent fusion with tumor cells within 12 h. In contrast, no internalization was observed in TM4SF1‐knockout Hepa1‐6 cells (Figure 2K). In summary, these results indicate that CAR.A18‐EVs specifically bind to human and murine TM4SF1 proteins both in vitro and at the cellular level. CAR.A18‐EVs exhibit excellent targeting ability and biocompatibility, establishing a solid foundation for their development as a low‐toxicity strategy for tumor immunotherapy.

### 2.5 Comparative Evaluation of TM4SF1 Nanobody‐ and scFv‐Based CAR Constructs

To further validate the rationale for employing a nanobody‐based CAR design, we directly compared the TM4SF1‐specific nanobody (Nb‐A18) with a conventional scFv fragment reported in the public patent (CN 117355333A). Nb‐A18 exhibited strong cross‐species recognition toward both human and murine TM4SF1 proteins, as confirmed by ELISA and SPR analyses, whereas the scFv fragment displayed limited cross‐reactivity (Figure S3A, Supporting Information). The nanobody also showed superior engineering efficiency due to its compact single‐domain architecture, which eliminates VH/VL mispairing and enables higher CAR surface expression in NKT cells compared with scFv‐based CARs (Figure S3B, Supporting Information). Moreover, Nb‐A18 demonstrated enhanced structural stability and functional persistence under prolonged antigen stimulation, maintaining over 90% CAR‐positive cells after three consecutive rechallenges, while scFv‐based CARs declined below 50% (Figure S3C, Supporting Information). In addition, CAR.A18‐EVs displayed markedly enhanced binding affinity for both human and murine TM4SF1 proteins compared with CAR.scFv‐EVs, Blank.NKT‐EVs, and CAR.ctrl‐EVs, as demonstrated by ELISA analysis (Figure S3D, Supporting Information). Furthermore, cell‐based ELISA confirmed that CAR.A18‐EVs specifically bound to TM4SF1‐high H22 and Hepa1‐6 cells, but not to TM4SF1–knockout Hepa1‐6 cells, indicating a high degree of target selectivity (Figure S3E, Supporting Information).

Collectively, these comparative results demonstrate that Nb‐A18 possesses superior molecular and functional properties compared with the scFv construct, including broader cross‐species recognition, higher expression efficiency, greater structural stability, and enhanced targeting capability, establishing it as an effective antigen‐recognition domain with translational potential for CAR‐based immunotherapy.

### 2.6 CAR^TM4SF1^‐EVs Exhibit Superior Tumor‐Targeting Capability and Safety

**Figure 3:**
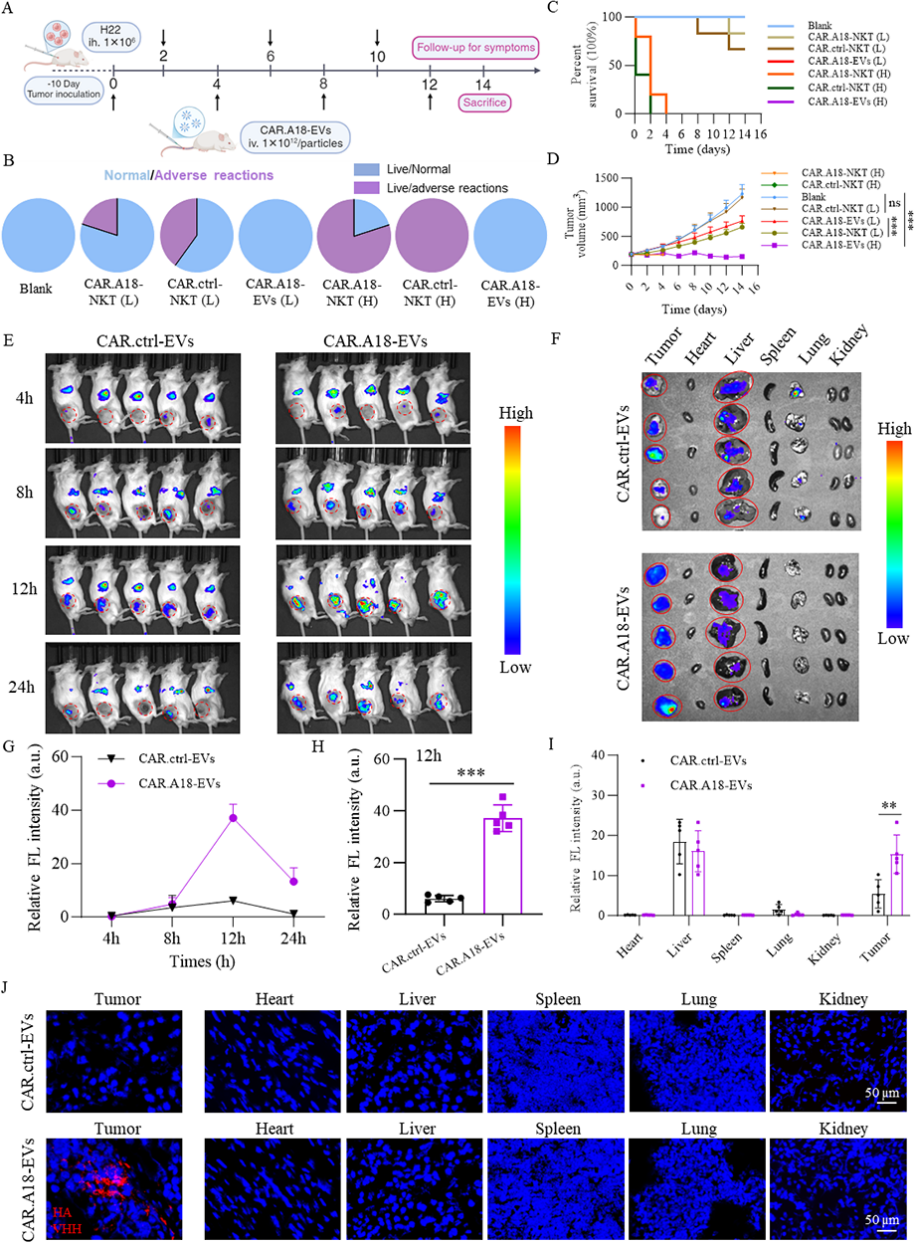
CAR^TM4SF1^‐EVs Derived from CAR^TM4SF1^‐NKT Cells Enhance Tumor Targeting and Safety. A) Schematic for treating subcutaneous HCC tumor‐bearing mice via intravenous injections of high or low doses of CAR.A18‐NKT cells (1 × 10^6^ or 1 × 10^5^ cells injection^−1^), CAR.ctrl‐NKT cells (1 × 10^6^ or 1 × 10^5^ cells injection^−1^), or CAR.A18‐EVs (1 × 10^12^ or 1 × 10^11^ particles injection^−1^). B) Statistical analysis of mice that exhibited adverse effects in each treatment group (*n* = 5). C) Survival analysis of mice treated with high or low doses of CAR.A18‐NKT cells, CAR.ctrl‐NKT cells, or CAR.A18‐EVs over the treatment period (*n* = 5). D) Tumor growth curves for mice treated with high or low doses of CAR.A18‐NKT cells, CAR.ctrl‐NKT cells, or CAR.A18‐EVs during the treatment period (*n* = 5). E) NIR‐I fluorescence imaging of subcutaneous H22 tumor‐bearing mice intravenously injected with CAR.ctrl‐EVs (1 × 10^11^ particles) or CAR.A18‐EVs (1 × 10^11^ particles) at 4, 8, 12, and 24 h post‐injection. F) NIR‐I fluorescence imaging of major organs from mice injected with CAR.Ctrl‐EVs or CAR.A18‐EVs at 24 h. G) Quantitative analysis of fluorescence intensity in the tumor region via NIR‐I imaging at different time points (panel E) (*n* = 5). H) Statistical analysis of fluorescence intensity in the tumor region from NIR‐I imaging at 12 h (panel E) (*n* = 5). I) Quantitative analysis of fluorescence intensity in major organs corresponding to Figure 3F (*n* = 5). The difference between imaging and quantitative results reflects local fluorescence intensity versus integrated organ signal, with liver values appearing higher due to its larger volume and broader EV distribution. J) IF staining of tumor tissues from subcutaneous HCC tumor‐bearing mice using anti‐HA and anti‐VHH antibodies to detect nanobodies. Scale bar, 50 µm. Quantitative data are presented as mean ± SD. Statistical significance was calculated using two‐way ANOVA (D, G, I) or one‐way ANOVA with Tukey's post‐test (H) or Mantel–Cox test (C). Statistical significance is indicated as ns (not significant), *p < 0.05, **p < 0.01, ***p < 0.001.

To ensure applicability and efficacy in vivo, an ideal nanotherapeutic agent must exhibit high biocompatibility and safety.^[^ ^36^ ^]^ We evaluated the safety and efficacy of CAR^4SF1^‐NKT cells and CAR^4SF1^‐EVs in a subcutaneous HCC (H22 cell line) mouse model. The mice were intravenously administered PBS (200 µL) or low‐dose CAR.A18‐NKT (1 × 10^5^ cells injection^−1^), low‐dose CAR.Ctrl‐NKT (1 × 10^5^ cells injection^−1^), low‐dose CAR.A18‐EVs (1 × 10^11^ particles injection^−1^), high‐dose CAR.A18‐NKT (1 × 10^6^ cells injection^−1^), high‐dose CAR.Ctrl‐NKT (1 × 10^6^ cells injection^−1^), or high‐dose CAR.A18‐EVs (1 × 10^12^ particles injection^−1^). Tumor volume, adverse reactions, and survival rates (with adverse reaction‐induced fatalities classified as deaths) were monitored (**Figure** **3**A). High‐dose CAR.A18‐NKT and CAR.ctrl‐NKT injections resulted in 100% mortality within 14 days of treatment and severe adverse effects within 4 days. Low‐dose CAR.A18‐NKT and CAR.ctrl‐NKT injections caused adverse effects in one and two mice, respectively. In contrast, both the low‐ and high‐dose CAR.A18‐EV groups, as well as the Blank group, exhibited no deaths or adverse effects throughout the treatment period (Figure 3B,C). These findings demonstrate the high safety profile of CAR.A18‐EVs, even after seven cycles of high‐dose injections. Although no adverse events were associated with the low‐dose CAR.A18‐EVs treatment, high‐dose CAR.A18‐EVs achieved the most significant tumor suppression compared to the other treatments (Figure 3D). These findings underscore the safety and efficacy of high‐dose CAR.A18‐EVs, highlighting their translational potential.

To further investigate the tumor‐targeting ability of CAR.A18‐EVs in vivo, DiR dye (1,1′‐Dioctadecyl‐3,3,3′,3′‐Tetramethylindotricarbocyanine iodide)‐labeled CAR.ctrl‐EVs and CAR.A18‐EVs were injected intravenously, and near‐infrared (NIR‐I) fluorescence imaging was conducted at 4, 8, 12, and 24 h (Figure 3E,F). At 4 and 8 h, no significant differences in fluorescence intensity were observed between CAR.A18‐EVs and CAR.ctrl‐EVs in the tumors (Figure 3G). However, at 12 h, CAR.A18‐EVs exhibited a significant peak in tumor accumulation, which was markedly greater than that of CAR.ctrl‐EVs (Figure 3H). By 24 h, the fluorescence intensity in the tumors had decreased, indicating efficient clearance of CAR.A18‐EVs, further supporting their safety. The biodistribution of CAR.A18‐EVs was assessed by sacrificing the mice at 24 h and performing NIR‐I imaging on major organs (heart, liver, spleen, lungs, and kidneys) and tumor tissues. Higher fluorescence signals were detected in the liver, suggesting that both CAR.A18‐EVs and CAR.ctrl‐EVs were metabolized primarily in the liver. Notably, tumor tissues in the CAR.A18‐EVs group showed significantly higher fluorescence signals compared to those in the CAR.ctrl‐EVs group (Figure 3F,I). To confirm the tissue distribution, IF staining with HA‐tag and anti‐VHH antibodies was performed on the major organs and tumor tissues. Compared to the that from the CAR.ctrl‐EVs group, tumor tissue from the CAR.A18‐EVs group displayed significantly increased nanobody fluorescence signals (Figure 3J). Minimal nanobody signals were detected in other organs, indicating the limited distribution or efficient clearance of CAR.A18‐EVs in nontumor tissues. In summary, CAR.A18‐EVs demonstrate excellent tumor‐targeting specificity and significant antitumor effects on subcutaneous HCC tumors. Additionally, repeated high‐dose intravenous injections of CAR.A18‐EVs are safe and effective in vivo.

### 2.7 CAR^TM4SF1^ Modification Enhances Tumor Specificity and Reduces Off‐Target Accumulation

To further clarify the biodistribution characteristics of CAR^4SF1^‐EVs, we next analyzed the in vivo localization of CAR^+^ and CAR^−^ EVs populations. Given that ≈60% of EVs express CAR molecules on their surface (Figure 2F), the remaining CAR^−^ EVs are more likely to undergo hepatic clearance via nonspecific uptake, while CAR^+^ EVs preferentially accumulate in TM4SF1‐high tumors. This explains why liver tissues show relatively high total fluorescence in biodistribution imaging but low CAR‐specific signal in immunofluorescence analysis.

To validate this hypothesis, we used immunoaffinity purification to isolate and purify CAR⁺ EVs from CAR^4SF1^‐NKT–derived EVs, followed by DiR labeling of both CAR.A18‐EVs and CAR.ctrl‐EVs for in vivo fluorescence imaging in Hepa1‐6 tumor–bearing mice. The results showed that CAR.A18‐EVs exhibited markedly enhanced tumor accumulation at 12 h (Figure S4A,C,D, Supporting Information), whereas their hepatic accumulation was significantly lower than that in tumors (Figure S4B,E, Supporting Information). In contrast, CAR.ctrl‐EVs displayed much higher nonspecific accumulation in the liver than in tumor tissues (Figure S4B,E, Supporting Information). These findings clearly demonstrate that CAR^4SF1^ modification markedly enhances tumor‐targeting specificity while minimizing off‐target hepatic uptake.

We further examined whether CAR.A18‐EVs exhibit nonspecific accumulation in normal cells. PKH67‐labeled CAR.A18‐EVs and CAR.ctrl‐EVs were injected into Hepa1‐6 tumor–bearing mice, followed by flow cytometry analysis of tumors, peripheral blood cells, and major organs (liver, kidney, and lung). The results showed that CAR.A18‐EVs displayed strong uptake by TM4SF1‐high Hepa1‐6 tumor cells but negligible accumulation in renal epithelial cells, pulmonary epithelial cells, or peripheral blood cells, while hepatic uptake was significantly lower than that of CAR.ctrl‐EVs (Figure S5, Supporting Information). These findings indicate that CAR^4SF1^ engineering confers enhanced tumor specificity and reduced nonspecific distribution in normal tissues.

Although low‐level TM4SF1 expression may exist in certain normal tissues, CAR.A18‐EVs exhibited minimal accumulation in these sites and did not induce observable toxicity. This favorable safety profile can be attributed to the improved tumor‐targeted delivery efficiency and reduced off‐target retention conferred by CAR^4SF1^ modification, which together effectively lower systemic exposure and potential toxicity risks.

Taken together, these results provide additional evidence that CAR.A18‐EVs achieve precise tumor‐targeted delivery with minimal off‐target retention, thereby ensuring excellent in vivo safety and supporting their translational potential for clinical application.

### 2.8 Pharmacokinetic Characterization of CAR^TM4SF1^‐EVs Using ⁸⁹Zr‐Based positron emission tomography Imaging

To further elucidate the in vivo pharmacokinetic profile and clearance dynamics of CAR^4SF1^‐EVs, we performed quantitative positron emission tomography (PET) imaging using ⁸⁹Zr radiolabeling, which provides superior sensitivity and tissue penetration compared with conventional fluorescence imaging or immunohistochemistry.^[^ ^37^, ^38^ ^]^ To avoid interference from nonspecific vesicles, EVs secreted by CAR^4SF1^‐NKT cells were isolated via ultracentrifugation, followed by immunoaffinity purification of CAR⁺ EVs. The purified CAR.A18‐EVs and CAR.ctrl‐EVs were then radiolabeled with ⁸⁹Zr using a DFO chelator and intravenously injected into Hepa1‐6 tumor–bearing mice. PET imaging and γ‐counting were performed at 2, 4, 8, 12, and 24 h post‐injection to systematically evaluate their biodistribution in tumors, blood, and major organs (heart, liver, spleen, lungs, and kidneys).

The results revealed that the radioactive signal of CAR.A18‐EVs in tumors progressively increased from 2 h, peaked at 12 h, and subsequently decreased by 24 h (Figure S6A, Supporting Information), indicating a time‐dependent enrichment and clearance pattern. In contrast, CAR.ctrl‐EVs displayed no significant tumor accumulation throughout the observation period, confirming the specific targeting capability of CAR.A18‐EVs.

In the bloodstream, both types of EVs were gradually cleared over time, with CAR.A18‐EVs exhibiting a faster clearance rate, suggesting more efficient trafficking from circulation to tumor sites (Figure S6B, Supporting Information). In non‐target organs, CAR.ctrl‐EVs showed persistent hepatic accumulation that remained high even at 24 h, consistent with nonspecific uptake by the reticuloendothelial system (Figure S6C, Supporting Information). Apart from this, both EV groups exhibited low distribution levels in other major organs, including the heart, spleen, lungs, and kidneys, with no significant differences (Figure S6D, Supporting Information). Further γ‐counting quantification at 24 h (% ID/g) demonstrated that CAR.A18‐EVs exhibited significantly higher % ID/g values in tumors compared with other organs, whereas CAR.ctrl‐EVs showed predominant retention in the liver with almost undetectable tumor signals (Figure S6E, Supporting Information).

Collectively, these data indicate that the signal increase observed between 8–12 h corresponds to the rapid tumor enrichment phase of CAR.A18‐EVs, followed by a gradual clearance process. Overall, CAR.A18‐EVs demonstrated efficient tumor‐targeted accumulation, rapid systemic clearance, and minimal nonspecific organ retention, underscoring their favorable pharmacokinetic properties, tumor specificity, and biosafety in vivo.

### 2.9 CAR^TM4SF1^‐EVs Safely and Effectively Inhibit Tumor Growth and Metastasis

**Figure 4:**
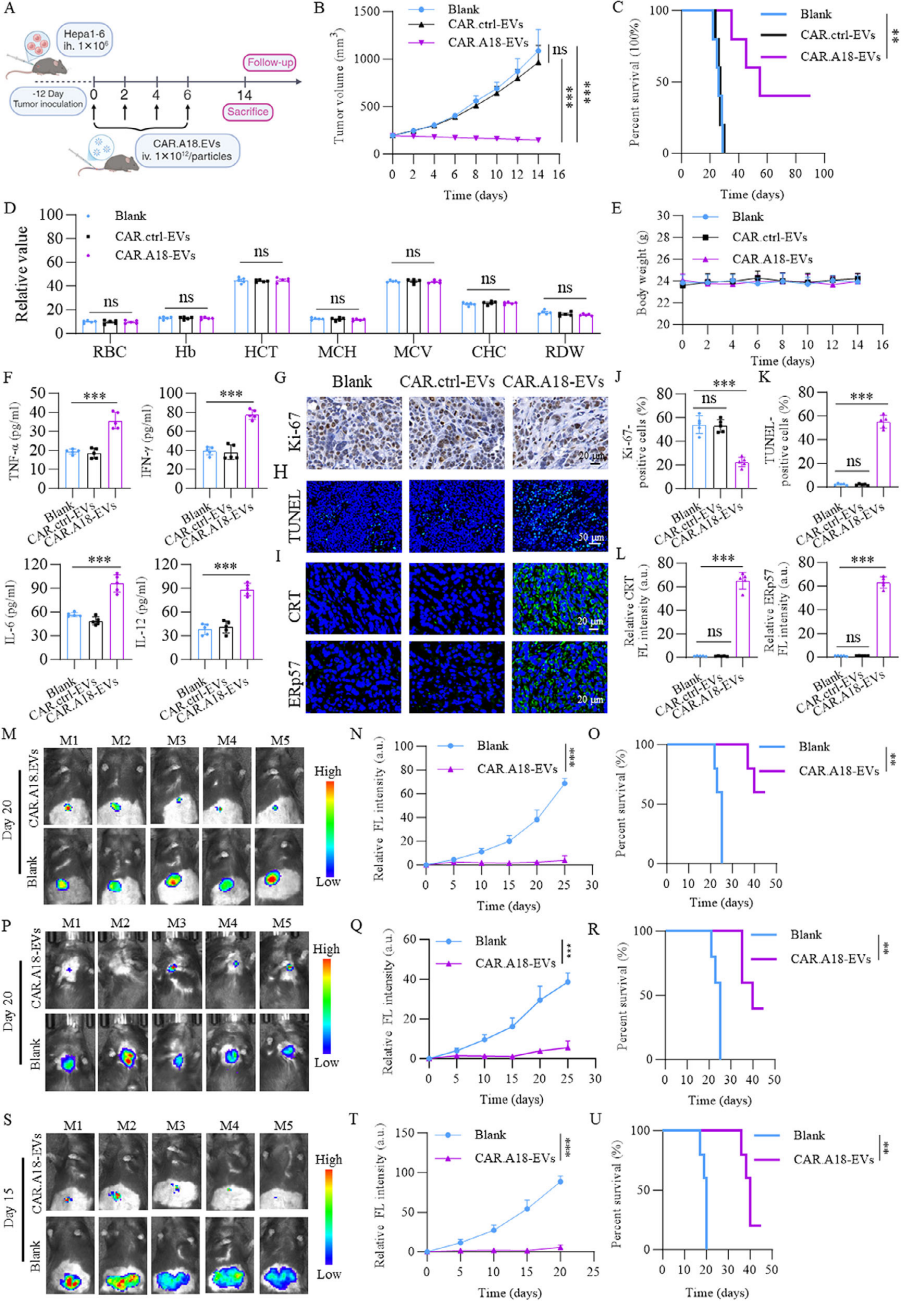
CAR^TM4SF1^‐EVs Significantly Inhibit Tumor Growth and Metastasis In Vivo. A) Schematic for treating subcutaneous Hepa1‐6 tumor‐bearing mice with PBS, CAR.ctrl‐EVs, or CAR.A18‐EVs via intravenous injection. B) Tumor growth curves for the mice in the Blank, CAR.ctrl‐EVs, and CAR.A18‐EVs groups (*n* = 5). C) Survival analysis of the mice in the Blank, CAR.ctrl‐EVs, and CAR.A18‐EVs groups (*n* = 5). D) CBC and biochemical tests for the mice in each group (*n* = 5). E) Body weight changes of the mice in each group during the treatment period (*n* = 5). F) Serum cytokine levels (TNF‐α, IFN‐γ, IL‐2, and IL‐6) in each group were measured by ELISA (*n* = 5). G) IHC analysis of Ki‐67 expression levels in tumor tissues from each group. H) IF staining of TUNEL‐stained tumor tissues from each group. I) IF analysis of CRT and ERp57 translocation in tumor tissues from each group. J) Quantification of Ki‐67 expression levels in tumor tissues (*n* = 5). K) Quantification of the percentage of TUNEL‐positive cells in tumor tissues (*n* = 5). L) Quantification of translocated CRT and ERp57 levels in tumor tissues (*n* = 5). M) Representative bioluminescence images of tumor signals in the lungs on day 20 from a lung metastasis model established by injecting Luc‐Hepa1‐6 cells into the tail vein and treating the mice with PBS or CAR.A18‐EVs (*n* = 5). N) Tumor signal dynamics in the lungs from bioluminescence imaging of the two groups (*n* = 5). O) Survival curves for mice in the lung metastasis model (*n* = 5). P) Representative bioluminescence images of tumor signals in the abdominal cavity on day 15 from an abdominal metastasis model established by injecting Luc‐Hepa1‐6 cells intraperitoneally and treating them with PBS or CAR.A18‐EVs (*n* = 5). Q) Tumor signal dynamics in the abdominal cavity from bioluminescence imaging of the two groups (*n* = 5). R) Survival curves for mice in the abdominal metastasis model (*n* = 5). S) Representative bioluminescence images of tumor signals in the liver on day 20 from a liver metastasis model established by injecting Luc‐Hepa1‐6 cells into the spleen of mice and treating them with PBS or CAR.A18‐EVs (*n* = 5). T) Tumor signal dynamics in the liver from bioluminescence imaging of the two groups (*n* = 5). U) Survival curves for mice in the liver metastasis model (*n* = 5). Quantitative data are presented as mean ± SD. Statistical significance was calculated using two‐way ANOVA (B, D, E, F, J, K, L, N, Q, T) with Tukey's post‐test or Mantel–Cox test (C, O, R, U). Statistical significance is indicated as ns (not significant), *p < 0.05, **p < 0.01, ***p < 0.001.

Given the excellent safety profile and tumor‐targeting efficacy of CAR.A18‐EVs, we further evaluated their therapeutic effects and safety in an HCC subcutaneous tumor model using Hepa1‐6 cell lines. Following the establishment of the Hepa1‐6 tumor mouse model, the mice were intravenously injected with PBS, CAR.ctrl‐EVs, or CAR.A18‐EVs four times, as outlined in **Figure** **4**A. Tumor volume analysis revealed that compared to the Blank and CAR.ctrl‐EVs groups, the CAR.A18‐EVs group presented the most significant reduction in tumor size (Figure 4B; Figure S7, Supporting Information). Additionally, CAR.A18‐EVs markedly extended survival over a 90‐day follow‐up period (Figure 4C). High biosafety is a critical prerequisite for the therapeutic application of nanomedicines. CRS, a severe immune response characterized by excessive cytokine release, is a major adverse event that is associated with CAR‐T‐cell therapies.^[^ ^33^ ^]^ To evaluate safety, we conducted detailed health assessments, including complete blood counts, body weight monitoring, cytokine profiling, and histological analysis. Blood analysis revealed no significant differences in key parameters, which were red blood cell (RBC) count, hemoglobin (Hb) levels, hematocrit (HCT) values, mean corpuscular hemoglobin (MCH) levels, mean corpuscular volume (MCV), complete blood count (CBC), and red blood cell distribution width (RDW), among the Blank, CAR.ctrl‐EVs, and CAR.A18‐EVs groups, with all values within normal ranges (Figure 4D). The body weights also remained stable across all the groups during treatment (Figure 4E). ELISA analysis of serum cytokines revealed increased levels of TNF‐α, IFN‐γ, IL‐2, and IL‐6 in the CAR.A18‐EVs group, indicating systemic immune activation without triggering CRS or related symptoms (Figure 4F). H&E staining analysis of major organs (heart, liver, spleen, lungs, and kidneys) revealed no structural abnormalities in any group (Figure S8, Supporting Information). These results demonstrate the safety of CAR.A18‐EVs in vivo. To investigate the antitumor mechanism of CAR.A18‐EVs, we performed IHC and IF staining on tumor tissues. Compared with Blank group and CAR.ctrl‐EVs, CAR.A18‐EVs significantly reduced Ki‐67 expression (Figure 4G,J) and increased TUNEL staining (Figure 4H,K), indicating that they suppressed proliferation and promoted apoptosis in tumor cells. Furthermore, the calreticulin (CRT) and ERp57 proteins translocated to the tumor cell surface in the CAR.A18‐EVs group compared with other groups, signifying the induction of ICD (Figure 4I,L).

Given the strong antitumor efficacy of CAR.A18‐EVs in the subcutaneous tumor mouse model, we examined their effects on HCC metastasis. Using a Luc‐Hepa1‐6 splenic injection liver metastasis model, we simulated clinical tumor cell dissemination via the portal vein. The mice were intravenously injected with PBS or CAR.A18‐EVs four times and imaged using bioluminescence. By day 20, compared to Blank group, CAR.A18‐EVs significantly reduced hepatic tumor signals (Figure 4M,N). Over a 45‐day follow‐up, CAR.A18‐EVs extended survival and cleared liver metastases in 60% of the mice (3/5) (Figure 4O). We also evaluated CAR.A18‐EVs in a Luc‐Hepa1‐6 lung metastasis model via tail vein injection. Bioluminescence imaging revealed reduced lung tumor signals in the CAR.A18‐EVs group (Figure 4P,Q), with a 40% complete clearance rate and prolonged survival over 45 days (2/5 mice) (Figure 4R). Finally, in a Luc‐Hepa1‐6 peritoneal metastasis model, the abdominal tumor signals were dramatically reduced in the CAR.A18‐EV group compared with the Blank group by day 15 (Figure 4S). Quantitative imaging analysis revealed significant differences in tumor signals, with extended survival and a 20% clearance rate of abdominal metastases in the CAR.A18‐EVs group (1/5 mice) (Figure 4T,U). These findings demonstrate that CAR.A18‐EVs effectively suppress tumor growth and metastasis by inducing ICD and systemic immune activation. Intravenous administration of CAR.A18‐EVs constitute a safe and promising therapeutic strategy for treating HCC.

### 2.10 Endoplasmic Reticulum Stress Plays a Central Role in CAR^TM4SF1^‐EVs–Mediated ICD

Endoplasmic reticulum (ER) stress is a well‐established upstream trigger and regulatory event in the induction of ICD.^[^ ^39^, ^40^ ^]^ During ICD, ER stress promotes the translocation of CRT and ERp57 to the cell surface and facilitates the release of damage‐associated molecular patterns (DAMPs) such as ATP and HMGB1, which collectively enhance dendritic cell activation and antitumor immunity.

Given these insights, we investigated whether ER stress contributes to the ICD phenotype induced by CAR.A18‐EVs. Treatment of tumor cells with CAR.A18‐EVs markedly activated ER stress, as evidenced by the upregulation of canonical markers ATF4, GRP94, and CHOP (Figure S9A, Supporting Information). This activation was accompanied by classical ICD hallmarks, including extracellular ATP and HMGB1 release and the surface exposure of CRT and ERp57 (Figure S9B–D, Supporting Information). In contrast, pharmacological inhibition of ER stress using 4‐phenylbutyric acid (4‐PBA) significantly attenuated these effects, resulting in reduced DAMP release and diminished CRT and ERp57 exposure (Figure S9E,F, Supporting Information).

Collectively, these findings indicate that CAR.A18‐EVs–induced ICD is critically dependent on ER stress activation, which functions as a key upstream signaling event linking EV‐mediated molecular delivery to immunogenic tumor cell death.

### 2.11 FasL as a Key Effector Mediating CAR^TM4SF1^‐EVs–Induced ICD

To further identify the functional molecules responsible for triggering ER stress–dependent ICD, we performed comparative quantitative proteomic analysis of EVs derived from resting NKT cells and activated CAR.A18‐NKT cells under equal particle number conditions. KEGG enrichment revealed that CAR.A18‐EVs cargo was significantly enriched in apoptosis, ER stress, and inflammatory signaling pathways, containing effector molecules such as FasL, Granzyme B, IL‐2, and CCL3, among others (Figure S10A, Supporting Information). Given the established role of FasL in regulating ER stress and apoptosis through the Fas/FasL axis,^[^ ^40^, ^41^ ^]^ we hypothesized that FasL might serve as a key mediator of CAR.A18‐EVs–induced ICD.

Because the activation state of NKT cells can profoundly influence the composition and biological function of the EVs they secrete, we next sought to verify that the CAR‐NKT cells used for EVs isolation were indeed in an activated state. Previous studies have reported that CD69 serves as an early activation marker and an important co‐stimulatory molecule regulating NKT cells proliferation and cytokine secretion.^[^ ^42^ ^]^ Therefore, we evaluated CD69 expression by flow cytometry, which revealed that both CAR.A18‐NKT and CAR.ctrl‐NKT cells displayed markedly elevated CD69 levels upon activation, confirming that the CAR‐NKT cells were in a fully activated state (Figure S10B, Supporting Information).

Having confirmed the activation status of CAR‐NKT cells, we next evaluated the impact of this activation on the molecular composition of their secreted EVs. We focused on representative effector molecules identified in the proteomic analysis. Western blot analysis showed that activated CAR.A18‐EVs exhibited markedly elevated FasL expression (Figure S10C, Supporting Information), accompanied by a significantly higher proportion of Granzyme B^+^ EVs as determined by nano‐flow cytometry (Figure S10D, Supporting Information). These findings indicate that activation of CAR‐NKT cells directly enhances the loading of functional effector proteins into EVs, thereby augmenting their downstream immunostimulatory potential.

To further validate the functional role of FasL, we generated FasL‐deficient CAR.A18‐NKT cells (Faslg‐knockout) and confirmed the absence of FasL protein in their secreted EVs (Figure S11A,B, Supporting Information). Compared with wild‐type EVs, FasL‐deficient CAR.A18‐EVs induced substantially lower expression of ER stress markers (ATF4, GRP94, CHOP) in tumor cells (Figure S11C, Supporting Information) and markedly reduced ICD hallmarks, including extracellular ATP and HMGB1 release and CRT exposure (Figure S11D,E, Supporting Information).

Collectively, these findings demonstrate that activation of CAR‐NKT cells enhances EV functionalization, and that FasL serves as a pivotal effector molecule enabling CAR.A18‐EVs to trigger ER stress–mediated ICD in TM4SF1‐high tumor cells, thereby establishing a mechanistic foundation for their subsequent immunostimulatory activity.

### 2.12 CAR^TM4SF1^‐EVs Remodel the Tumor Immune Microenvironment and Rely on CD8^+^ T Cells for Potent Antitumor Immunity

**Figure 5:**
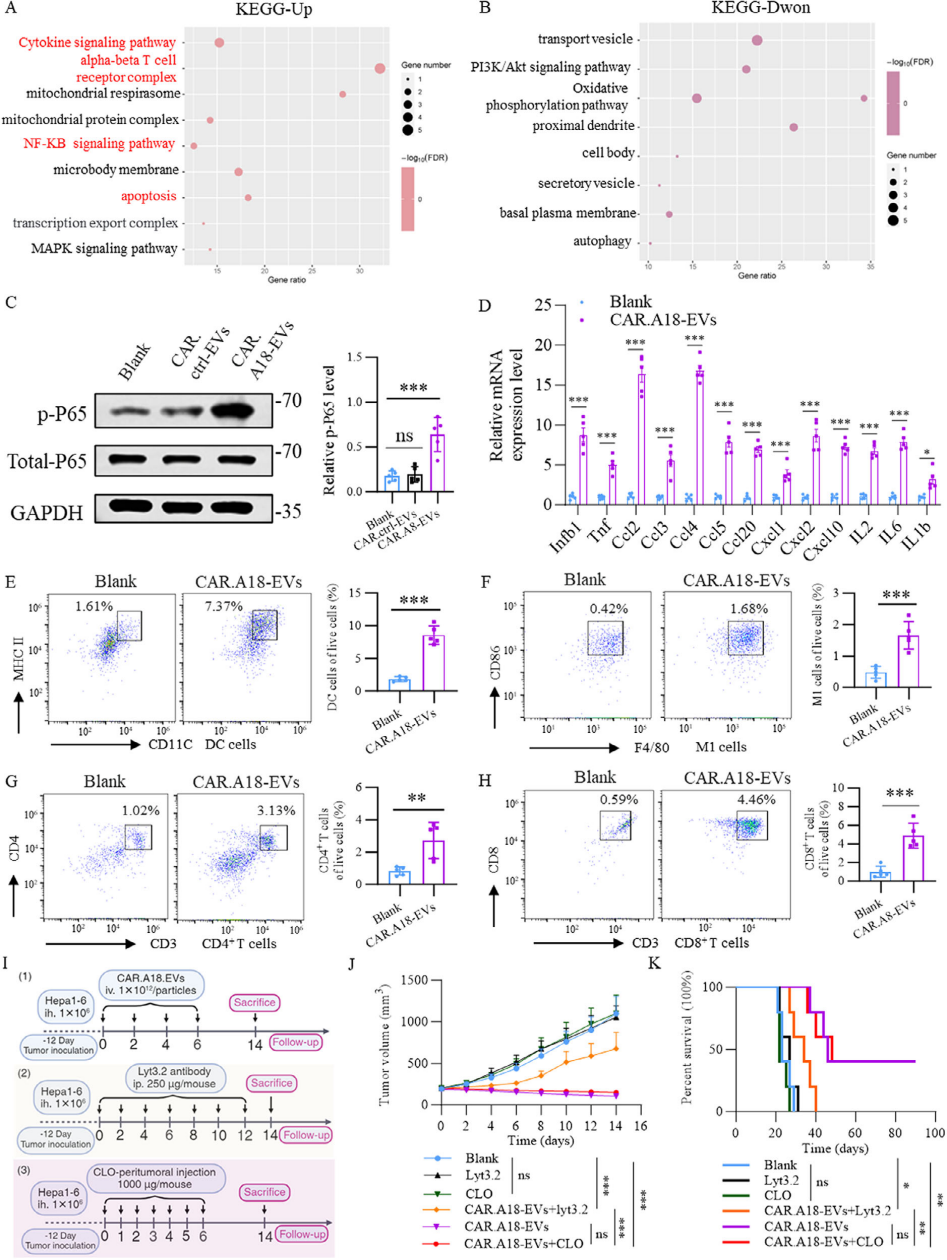
CAR^TM4SF1^‐EVs Depend on CD8^+^ T Cells to Exert Their Exceptional Antitumor Effects In Vivo. A) KEGG pathway enrichment analysis of the upregulated genes identified by RNA‐seq in tumor tissues from the Blank and CAR.A18‐EVs groups. B) KEGG pathway enrichment analysis of the downregulated genes identified by RNA‐seq in tumor tissues from the Blank and CAR.A18‐EVs groups (*n* = 5). C) Western blot analysis of total P65 and P‐P65 levels in tumor tissues from the Blank, CAR.ctrl‐EV, and CAR.A18‐EVs groups (*n* = 5). D) qRT‒PCR quantification of cytokine expression levels in tumor tissues from the Blank and CAR.A18‐EVs groups (*n* = 5). E) Flow cytometry analysis of mature dendritic cells (CD45^+^ CD11c^+^ MHC II^+^) in tumor tissues from the Blank and CAR.A18‐EVs groups (*n* = 5). F) Flow cytometry analysis of M1 macrophages (CD45^+^ F4/80^+^ CD86^+^) in tumor tissues from the Blank and CAR.A18‐EVs groups (*n* = 5). G) Flow cytometry analysis of CD4^+^ T cells (CD45^+^ CD3^+^ CD4^+^) in tumor tissues from the Blank and CAR.A18‐EVs groups (*n* = 5). H) Flow cytometry analysis of CD8^+^ T cells (CD45^+^ CD3^+^ CD8^+^) in tumor tissues from the Blank and CAR.A18‐EVs groups (*n* = 5). I) Schematic illustrating the treatment of subcutaneous Hepa1‐6 tumors with CAR.A18‐EVs combined with CD8^+^ T‐cell or macrophage depletion. J) Tumor growth curves during treatment across six groups of mice: PBS, Lyt3.2 (i.p. 250 µg mouse^−1^, every other day), CLO (peritumoral injection daily, 1000 µg mouse^−1^), CAR.A18‐EVs (1 × 10^12^ particles injection^−1^), CAR.A18‐EVs (1 × 10^12^ particles injection^−1^) + Lyt3.2 (i.p. 250 µg mouse^−1^, every other day), and CAR.A18‐EVs (1 × 10^12^ particles injection^−1^) + CLO (peritumoral injection daily, 1000 µg mouse^−1^) (*n* = 5). K) Survival curves for the six groups of mice during the treatment period (*n* = 5). Quantitative data are presented as mean ± SD. Statistical significance was calculated using one‐way ANOVA with Tukey's post‐test (D, E‐H) or two‐way ANOVA with Tukey's post‐test (J) or Mantel–Cox test (K). Statistical significance is indicated as ns (not significant), *p < 0.05, **p < 0.01, ***p < 0.001.

Given that CAR.A18‐EVs can induce ICD in tumor cells and potentially promote systemic immune activation, we performed RNA sequencing (RNA‐seq) on tumor tissues from mice in the Blank group and the CAR.A18‐EVs group to investigate the molecular mechanisms underlying the antitumor effects of CAR.A18‐EVs. A total of 161 significantly differentially expressed genes were identified, and heatmap analysis revealed distinct expression patterns between the two groups (Figure S12A,B, Supporting Information). Kyoto Encyclopedia of Genes and Genomes (KEGG) pathway enrichment analysis revealed that the cytokine signaling pathway, alpha‐beta T‐cell receptor complex, NF‐κB signaling pathway, and apoptosis pathway were enriched primarily in the upregulated genes (**Figure** **5**A), whereas the oxidative phosphorylation (OXPHOS) pathway was enriched in the downregulated genes (Figure 5B). The cytokine signaling pathway plays a central role in immune responses by regulating immune cell activation, proliferation, and differentiation through cytokines such as interferons (IFNs) and interleukins (ILs).^[^ ^43^ ^]^ The alpha‐beta T‐cell receptor complex mediates T‐cell recognition of antigen peptide‐MHC complexes, initiating adaptive immune responses,^[^ ^44^, ^45^ ^]^ whereas the NF‐κB signaling pathway modulates lymphocyte activation, cytokine production, and inflammation regulation.^[^ ^46^, ^47^ ^]^ The apoptosis pathway is essential for immune cell development, tolerance, and tumor cell clearance because it balances cell survival and death.^[^ ^48^, ^49^ ^]^ Collectively, these pathways highlight the critical role of CAR.A18‐EVs in promoting immune activation and tumor suppression.

To validate the RNA‐seq findings, Western blot analysis revealed a significant increase in phosphorylated P65 (p‐P65) levels, indicating robust activation of the NF‐κB signaling pathway in the CAR.A18‐EV group compared with the Blank and CAR.ctrl‐EVs groups (Figure 5C). Consistent with immune activation, qRT‒PCR analysis revealed elevated expression of key cytokines and chemokines, including *Infb1* (type I interferon), *Tnf* (proinflammatory cytokine), CC chemokines (*Ccl2*, *Ccl3*, *Ccl4*, *Ccl5*, and *Ccl20*), CXC chemokines (*Cxcl1*, *Cxcl2*, and *Cxcl10*), and interleukins (*Il2*, *Il6*, and *Il1b*), further confirming systemic immune activation (Figure 5D). Flow cytometry analysis revealed significantly increased infiltration of mature dendritic cells (CD45^+^ CD11c^+^ MHC II^+^), M1 macrophages (CD45^+^ F4/80^+^ CD86^+^), CD4^+^ T cells (CD45^+^ CD3^+^ CD4^+^), and CD8^+^ T cells (CD45^+^ CD3^+^ CD8^+^) in the tumor microenvironment of the CAR.A18‐EVs group compared to the Blank group, with fold increases of 4.7, 3.4, 3.4, and 4.8, respectively. Concurrently, we observed no significant changes in M2 macrophage (CD45^+^ F4/80^+^ CD206^+^) populations (Figure 5E–H; Figure S13A,B, Supporting Information). These results demonstrate that CAR.A18‐EVs induce substantial immune cell infiltration into the tumor microenvironment and effectively enhance antitumor immunity.

To further delineate the immune mechanisms underlying CAR.A18‐EV–mediated tumor inhibition, we employed immune cell depletion strategies. The Lyt3.2 antibody, a monoclonal antibody targeting CD8β, specifically binds to and depletes CD8^+^ T cells through intraperitoneal injection, making it an effective tool for studying the role of CD8^+^ T cells in vivo.^[^ ^50^ ^]^ Similarly, clodronate liposome (CLO) is a macrophage depletion agent that selectively eliminates macrophages without affecting other cell types, making it widely used to investigate macrophage functions in the tumor microenvironment.^[^ ^51^ ^]^ Considering that CAR.A18‐EVs increased the abundance of CD8^+^ T cells and M1 macrophages in the tumor microenvironment, we aimed to determine the primary immune cell population responsible for the antitumor effects of CAR.A18‐EVs. CD8^+^ T cells and macrophages were selectively depleted in Hepa1‐6 subcutaneous tumor‐bearing mice using the Lyt3.2 antibody or CLO, respectively.

As illustrated in Figure 5I, Hepa1‐6 tumor‐bearing mice received injections of PBS (200 µL), Lyt3.2 antibody (i.p. 250 µg mouse^−1^, every other day), CLO (peritumoral injection daily, 1000 µg mouse^−1^), CAR.A18‐EVs (1 × 10^12^ particles injection^−1^), CAR.A18‐EVs (1 × 10^12^ particles injection^−1^) + Lyt3.2 antibody (i.p. 250 µg mouse^−1^, every other day), and CAR.A18‐EVs (1 × 10^12^ particles injection^−1^) + CLO (peritumoral injection daily, 1000 µg mouse^−1^). The results showed no significant differences in tumor volume among the Blank, Lyt3.2, and CLO groups (Figure 5J). In accordance with the experimental design (Figure 5I), the mice received injections of PBS (200 µL), the Lyt3.2 antibody (intraperitoneal (i.p.), 250 µg mouse^−1^, every other day), CLO (peritumoral injection daily, 1000 µg mouse^−1^), CAR.A18‐EVs (1 × 10^12^ particles injection^−1^), CAR.A18‐EVs (1 × 10^12^ particles injection^−1^)+Lyt3.2 antibody (i.p. 250 µg mouse^−1^, every other day), or CAR.A18‐EVs (1 × 10^12^ particles injection^−1^)+CLO (peritumoral injection daily, 1000 µg mouse^−1^). The results revealed no significant differences in tumor volume among the Blank, Lyt3.2, and CLO groups (Figure 5J). Moreover, when CAR.A18‐EVs were combined with CLO, tumor suppression was comparable to that observed with CAR.A18‐EVs alone, with no significant difference in tumor volume between these two groups. Interestingly, combining CAR.A18‐EVs with the Lyt3.2 antibody partially abrogated the antitumor effects of CAR.A18‐EVs. Although the tumor volume in the CAR.A18‐EVs+Lyt3.2 group was significantly smaller than that in the Blank, Lyt3.2, and CLO groups, it was notably larger than that in the CAR.A18‐EVs+CLO and CAR.A18‐EVs groups.

Survival analysis further corroborated these findings: the CAR.A18‐EVs + Lyt3.2 group exhibited extended survival compared with the Blank, Lyt3.2, and CLO groups, but significantly shorter survival than the CAR.A18‐EVs and CAR.A18‐EVs + CLO groups (Figure 5K). Collectively, these results demonstrate that macrophage depletion does not significantly affect the therapeutic efficacy of CAR.A18‐EVs, whereas CD8⁺ T‐cell depletion partially compromises their antitumor effects.

In conclusion, CAR.A18‐EVs activate NF‐κB signaling to induce systemic antitumor immune responses, promoting dendritic cell maturation and the infiltration of various immune cells into the tumor microenvironment. The exceptional antitumor efficacy of CAR.A18‐EVs is mediated primarily by CD8^+^ T cells, highlighting their pivotal role in the immune response triggered by this therapeutic strategy.

### 2.13 ER Stress–Mediated ICD Serves as the Primary Mechanism Underlying the Antitumor Activity of CAR^TM4SF1^‐EVs

Given that transcriptomic analysis (Figure 5B) revealed a significant downregulation of OXPHOS pathways following CAR.A18‐EV treatment, we next sought to further elucidate the mechanistic basis underlying the potent antitumor effects of CAR^4SF1^‐EVs by examining the relative contributions of OXPHOS and ER stress to their therapeutic efficacy. A six‐group comparative study was conducted in a subcutaneous Hepa1‐6 tumor–bearing mouse model, as outlined in Figure S14A (Supporting Information): 1) Blank group (PBS, 200 µL); 2) CAR.ctrl‐EVs group (1 × 10^12^ particles injection^−1^); 3) CAR.A18‐EVs group (1 × 10^12^ particles injection^−1^); 4) Rotenone group (OXPHOS inhibitor, 2 mg/kg/day, i.p.): Rotenone, a classical mitochondrial complex I inhibitor, blocks the electron transport chain and suppresses oxidative phosphorylation.^[^ ^52^ ^]^ 5) CAR.A18‐EVs (1 × 10^12^ particles injection^−1^) + Dichloroacetate (DCA, OXPHOS activator dichloroacetate, 50 mg/kg/day, i.p.) group: DCA, a pyruvate dehydrogenase kinase (PDK) inhibitor that enhances mitochondrial oxidative metabolism;^[^ ^53^ ^]^ and 6) CAR.A18‐EVs (1 × 10^12^ particles injection^−1^) + 4‐PBA (1 g/kg/day, p.o.) group. Tumor volume, survival, and individual tumor growth curves were monitored across all treatment groups. The results showed that CAR.A18‐EVs treatment resulted in marked tumor suppression and prolonged survival compared with the control groups (Figure S14B–D, Supporting Information). However, co‐administration of 4‐PBA significantly diminished the antitumor efficacy and survival benefit of CAR.A18‐EVs, indicating that their therapeutic effect critically depends on the activation of the ER stress pathway and subsequent induction of ICD. In contrast, Rotenone monotherapy produced only mild inhibition of tumor growth, without a significant difference compared with the Blank group, suggesting that OXPHOS inhibition alone is insufficient to drive robust tumor suppression. Moreover, OXPHOS upregulation by DCA did not notably compromise the efficacy of CAR.A18‐EVs, indicating that modulation of oxidative metabolism plays only a supportive rather than a primary role in their antitumor mechanism.

Taken together, these results demonstrate that the dominant mechanism by which CAR.A18‐EVs exert antitumor effects is through ER stress–mediated ICD induction, whereas OXPHOS downregulation likely serves as an auxiliary metabolic adaptation that facilitates, but does not determine, therapeutic efficacy.

### 2.14 CAR^TM4SF1^‐EVs Increase the Efficacy of Immune Checkpoint Blockade Therapy and Induce Long‐Lasting Antitumor Immune Memory

**Figure 6:**
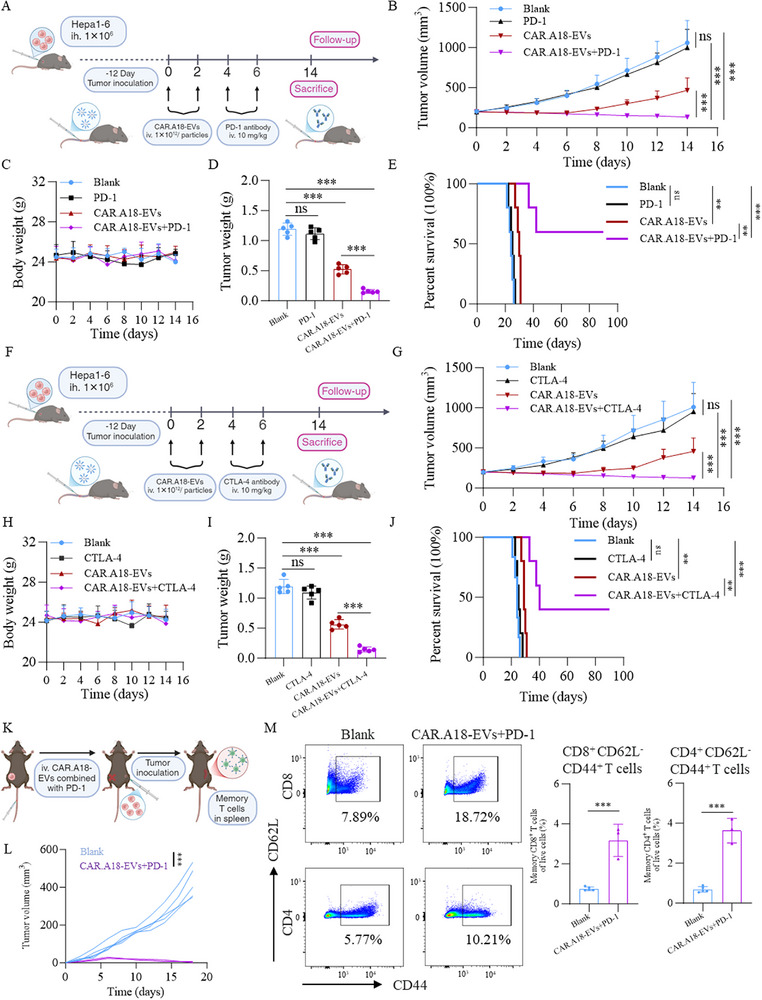
CAR^TM4SF1^‐EVs Combined with ICB Therapy Enhances Antitumor Efficacy and Induces Long‐Lasting Antitumor Immune Memory. A) Schematic of the treatment of subcutaneous Hepa1‐6 tumors with CAR.A18‐EVs combined with an anti‐PD‐1 antibody. B) Tumor growth curves of the four groups (Blank (200 µL, PBS), PD‐1 antibody (i.v. 10 mg kg^−1^), CAR.A18‐EVs (1 × 10^12^ particles injection^−1^), and CAR.A18‐EVs (1 × 10^12^ particles injection^−1^) + anti‐PD‐1 antibody (i.v. 10 mg kg^−1^)) during treatment (*n* = 5). C) Body weight measurements of the four groups over time (*n* = 5). D) Tumor weights of the four groups were measured on day 14 of treatment (*n* = 5). E) Survival curves of the four groups during the treatment period (*n* = 5). F) Schematic workflow for the treatment of subcutaneous Hepa1‐6 tumors using CAR.A18‐EVs combined with an anti‐CTLA‐4 antibody. G) Tumor growth curves of the four groups (Blank (200 µL, PBS), CTLA‐4 antibody (i.v. 10 mg kg^−1^), CAR.A18‐EVs (1 × 10^12^ particles injection^−1^), and CAR.A18‐EVs (1 × 10^12^ particles injection^−1^) + anti‐CTLA‐4 antibody (i.v. 10 mg kg^−1^)) during treatment (*n* = 5). H) Body weight measurements of the four groups over time (*n* = 5). I) Tumor weights of the four groups were measured on day 14 of treatment (*n* = 5). J) Survival curves of the four groups during the treatment period (*n* = 5). K) Schematic workflow for rechallenging mice that achieved complete remission after CAR.A18‐EV and anti‐PD‐1 antibody treatment, alongside Blank mice, with subcutaneous Hepa1‐6 tumor cells. Memory T cells in the spleen were analyzed on day 18. L) Tumor growth curves of the two groups (Blank and CAR.A18‐EVs + anti‐PD‐1 antibody) after rechallenge. M) Flow cytometry analysis of CD4^+^ memory T cells (CD4^+^ CD62L^−^ CD44^+^) and CD8^+^ memory T cells (CD8^+^ CD62L^−^ CD44^+^) in the spleens of the two groups. Quantitative data are presented as mean ± SD. Statistical significance was calculated using two‐way ANOVA (B, C, G, H, L) or one‐way ANOVA with Tukey's post‐test (D, I, M) or Mantel–Cox test (E, J). Statistical significance is indicated as ns (not significant), *p < 0.05, **p < 0.01, ***p < 0.001.

Immune checkpoint inhibitors, such as PD‐1 and CTLA‐4 antibodies, are emerging therapies for HCC that restore the ability of the immune system to target and eliminate tumors by blocking immune evasion pathways.^[^ ^54^ ^]^ Anti‐PD‐1 antibodies block the interaction between PD‐1 and PD‐L1, reactivating T‐cell‐mediated antitumor immunity,^[^ ^55^ ^]^ whereas anti‐CTLA‐4 antibodies promote T‐cell activation and proliferation by blocking the binding of CTLA‐4 to its ligands CD80/CD86.^[^ ^56^ ^]^ However, the immunosuppressive tumor microenvironment in HCC significantly limits the therapeutic efficacy of these agents. Since CAR.A18‐EVs can reprogram the immunosuppressive TME and increase CD4^+^ and CD8^+^ T‐cell populations, we investigated their ability to increase the efficacy of anti‐PD‐1 and anti‐CTLA‐4 antibodies. In a subcutaneous Hepa1‐6 tumor model, mice were intravenously injected twice with PBS (200 µL), an anti‐PD‐1 antibody (i.v., 10 mg/kg), CAR.A18‐EVs (1 × 10^12^ particles injection^−1^), or a combination of an anti‐PD‐1 antibody (i.v., 10 mg kg^−1^) and CAR.A18‐EVs (1 × 10^12^ particles injection^−1^) (**Figure** **6**A). Tumor growth analysis (Figure 6B; Figure S15, Supporting Information) revealed no significant differences between the Blank and anti‐PD‐1 antibody groups. While CAR.A18‐EVs alone initially suppressed tumor growth, tumor size rapidly increased upon cessation of treatment. In contrast, the combination of CAR.A18‐EVs and the anti‐PD‐1 antibody resulted in sustained tumor suppression, with significantly lower tumor volume and weight in the combination treatment group than the Blank, anti‐PD‐1 antibody, and CAR.A18‐EVs groups (Figure 6D). Furthermore, CAR.A18‐EVs alone moderately prolonged mouse survival, whereas the combination therapy significantly prolonged survival (Figure 6E). The results of the safety analysis revealed no abnormalities in body weight (Figure 6C), blood count (Figure S16, Supporting Information), or histological analysis of major organs (Figure S17, Supporting Information).

In addition, we further explored whether CAR.A18‐EVs could increase the efficacy of anti‐CTLA‐4 antibodies. Similarly, in a subcutaneous Hepa1‐6 tumor model, mice were intravenously injected twice with PBS (200 µL), an anti‐CTLA‐4 antibody (i.v., 10 mg kg^−1^), CAR.A18‐EVs (1 × 10^12^ particles injection^−1^), or a combination of an anti‐CTLA‐4 antibody (i.v., 10 mg kg^−1^) and CAR.A18‐EVs (1 × 10^12^ particles injection^−1^) (Figure 6F). Tumor volume analysis revealed no significant reduction in the anti‐CTLA‐4 antibody group (Figure 6G; Figure S18, Supporting Information). However, the combination of CAR.A18‐EVs and the anti‐CTLA‐4 antibody led to sustained tumor suppression, with significant reductions in tumor volume and weight compared to the Blank, anti‐CTLA‐4 antibody, and CAR.A18‐EVs groups (Figure 6I). The combination therapy also significantly prolonged mouse survival (Figure 6J), and no abnormalities in body weight (Figure 6H), blood count (Figure S19, Supporting Information), or major organ histology (Figure S20, Supporting Information) were detected, confirming the safety of this strategy. CAR.A18‐EVs synergized with both anti‐PD‐1 and anti‐CTLA‐4 antibodies to increase antitumor efficacy and overcome the limitations of immune checkpoint blockade in HCC. These results demonstrate that CAR.A18‐EVs combined with ICB therapy constitute a safe and effective immunotherapeutic strategy, offering potential for improved treatment outcomes and long‐lasting antitumor immune memory.

Recent studies have reported that immune memory T cells play a pivotal role in sustaining long‐term protection following complete tumor eradication.^[^ ^57^ ^]^ These cells not only persist in the body to prevent tumor recurrence but also respond swiftly and robustly upon re‐exposure to the same tumor antigen, ensuring rapid and potent immune responses.^[^ ^58^ ^]^ Owing to their specificity for tumor cells, immune memory T cells precisely target malignant cells without harming healthy tissues, contributing significantly to the rejection of tumor cell rechallenge. Considering that CAR.A18‐EVs combined with an anti‐PD‐1 antibody achieved complete tumor clearance in 60% (3/5) of the treated mice, we investigated whether this combination therapy promoted the generation of immune memory T cells in vivo.

According to the experimental design, the three mice that achieved complete remission were rechallenged subcutaneously with Hepa1‐6 cells along with five naïve mice as controls (Figure 6K). Tumor growth was closely monitored. As shown in our results, no significant tumor growth was observed in the CAR.A18‐EVs and anti‐PD‐1 antibody group compared to the Blank group, in which tumors formed readily (Figure 6L). Subsequent flow cytometry analysis of splenocytes revealed a marked increase in the percentages of CD4^+^ effector memory T cells (CD4^+^ CD62L^−^ CD44^+^) and CD8^+^ effector memory T cells (CD8^+^ CD62L^−^ CD44^+^) in the CAR.A18‐EVs + anti‐PD‐1 antibody group compared to the Blank group (Figure 6M). These memory T cells are known to play essential roles not only in initial immune responses but also in long‐term immune protection and antitumor immunity.^[^ ^59^ ^]^

Collectively, CAR.A18‐EVs remodel the immunosuppressive microenvironment of HCC, enhance immune checkpoint blockade efficacy, and induce robust, durable immune memory. The combination of CAR.A18‐EVs with PD‐1 or CTLA‐4 antibody represents a promising, safe, and clinically translatable strategy for long‐lasting tumor control.

Finally, several limitations should be acknowledged. Although in vitro expansion of CAR‐NKT cells can increase EV yield, scalable production systems for clinical‐grade CAR‐EVs need to be established. In addition, continuous optimization of CAR.A18‐EVs dosing will be critical to further improving their safety profile. Future studies exploring their antitumor efficacy across multiple tumor models will provide stronger preclinical evidence and further support their clinical translational potential.

## 3 Conclusion

In conclusion, our findings demonstrate that CAR.A18‐EVs represent a promising cancer therapeutic approach, effectively avoiding the adverse effects associated with CAR‐NKT cell therapy while significantly reshaping the immunosuppressive microenvironment of HCC, inhibiting tumor growth and metastasis, and improving OS. Furthermore, CAR.A18‐EVs increase the efficacy of ICB therapy, and their combination induces robust and long‐lasting antitumor immune memory. This safe and effective strategy holds great potential as a next‐generation treatment for targeted cancer immunotherapy.

## 4 Experimental Section

### Human Subjects

All human samples used in this study were obtained from patients pathologically diagnosed with primary HCC. Specifically, 36 paired tumor and adjacent non‐tumorous tissues were collected. All procedures involving human participants were approved by the Ethics Committees of Nanjing Medical University (2019‐SRFA‐238) and Zhengzhou University (2019‐SRFA‐238). All participants in this study obtained informed written consent from themselves or their close relatives.

### Animals and Experimental Procedures

All mouse experiments were conducted in accordance with protocols approved by the Institutional Animal Care and Use Committee (IACUC) of the University of Chicago (71829‐11) and the Nanjing Medical University Animal Management Committee (IACUC‐2404099), in compliance with institutional and national guidelines and regulations. Immunocompetent C57BL/6 mice and BALB/c mice were obtained from The Jackson Laboratory. Mice used in in vivo experiments were between 6 and 8 weeks of age, with equal numbers of male and female mice included in each experimental group. Animals were housed under standard institutional conditions, and group sizes are detailed in the figure legends.

### Cell Lines and Cell Culture

The Hepa1‐6 (RRID: CVCL_0327) murine HCC cell line was obtained from the American Type Culture Collection (ATCC, Manassas, VA, USA; purchased in 2022). The H22 (RRID: CVCL_H613) murine HCC cell line and HEK‐293T (RRID: CVCL_0063) cell line were obtained from Fuheng Biology (Shanghai, China; purchased in 2023). Hepa1‐6 and HEK‐293T cells were maintained in high‐glucose Dulbecco's Modified Eagle Medium (DMEM; Gibco, USA), whereas H22 cells were cultured in RPMI‐1640 medium (Gibco, USA). All media were supplemented with 10% FBS and 2 mM L‐glutamine (Gibco, USA). All cell lines were cultured at 37 °C in a humidified atmosphere containing 5% CO_2_. All cell lines were authenticated by short tandem repeat (STR) profiling prior to use. Mycoplasma contamination was routinely tested using a Mycoplasma Detection Kit (Lonza, USA), and all cell lines were confirmed to be mycoplasma‐free. None of the cell lines used in this study appear on the ICLAC Register of Misidentified Cell Lines. Detailed methods were available in the Supporting Information.

### Statistical Analysis

No data pre‐processing (including data transformation, normalization, or outlier removal) was performed prior to statistical testing. Unless otherwise specified, data are presented as the mean ± SD. Sample sizes (*n*) are provided in each figure legend. Differences between two samples were analyzed using an unpaired two‐tailed Student's t‐test. One‐way ANOVA was employed to compare tumor weights and various toxicological parameters among four groups, while two‐way ANOVA was used to assess in vitro cell viability and tumor growth curves. Survival curves were compared using log‐rank tests. A *p*‐value of less than 0.05 was considered statistically significant. Asterisks indicate significant differences (*p < 0.05, **p < 0.01, ***p < 0.001). Statistical analyses were performed using GraphPad Prism 10 software.

## Conflict of Interest

The authors declare no conflict of interest.

## Author Contributions

X.P.H., C.M.Q., Y.Z.Z., X.Q.W., and X.J.Q. contributed equally to this work and are co‐first authors. P.X., W.W.T., H.Z. and J.X.Z. conceived and designed the experiments. X.P.H., C.M.Q., Y.Z.Z., X.Q.W., and X.J.Q. conducted the database searches and bioinformatics analysis. X.P.H., C.M.Q., Y.Z.Z., X.Q.W., and X.J.Q., X.C., F.H., X.K.Z., Y.Y.J., H.L., and C.W.J. performed the experiments. X.P.H. and C.M.Q. wrote the paper. P.X., W.W.T., H.Z., and J.X.Z. participated in the revision of the draft. All authors read and approved the final paper.

## Supporting information



Supporting Information

## Data Availability

The data that support the findings of this study are available from the corresponding author upon reasonable request.

## References

[1] D. J. Baker , Z. Arany , J. A. Baur , J. A. Epstein , C. H. June , Nature 2023, 619, 707. 37495877 10.1038/s41586-023-06243-w PMC12522170

[2] J. N. Brudno , M. V. Maus , C. S. Hinrichs , JAMA, J. Am. Med. Assoc. 2024, 332, 1924. 10.1001/jama.2024.19462 PMC11808657 39495525

[3] C. L. Flugel , R. G. Majzner , G. Krenciute , G. Dotti , S. R. Riddell , D. L. Wagner , M. Abou‐El‐Enein , Nat. Rev. Clin. Oncol. 2023, 20, 49. 36418477 10.1038/s41571-022-00704-3 PMC10278599

[4] S. M. Albelda , Nat. Rev. Clin. Oncol. 2024, 21, 47. 37904019 10.1038/s41571-023-00832-4

[5] J. Wagner , E. Wickman , C. DeRenzo , S. Gottschalk , Mol. Ther. 2020, 28, 2320. 32979309 10.1016/j.ymthe.2020.09.015 PMC7647674

[6] F. Jacobsen , R. Pushpadevan , F. Viehweger , M. Freytag , R. Schlichter , N. Gorbokon , F. Buscheck , A. M. Luebke , D. Putri , M. Kluth , C. Hube‐Magg , A. Hinsch , D. Hoflmayer , C. Fraune , C. Bernreuther , P. Lebok , G. Sauter , S. Minner , S. Steurer , R. Simon , E. Burandt , D. Dum , F. Lutz , A. H. Marx , T. Krech , T. S. Clauditz , Pathol. Res. Pract. 2024, 256, 155175. 38452580 10.1016/j.prp.2024.155175

[7] A. Heczey , A. N. Courtney , A. Montalbano , S. Robinson , K. Liu , M. Li , N. Ghatwai , O. Dakhova , B. Liu , T. Raveh‐Sadka , C. N. Chauvin‐Fleurence , X. Xu , H. Ngai , E. J. Di Pierro , B. Savoldo , G. Dotti , L. S. Metelitsa , Nat. Med. 2020, 26, 1686. 33046868 10.1038/s41591-020-1074-2

[8] A. Heczey , X. Xu , A. N. Courtney , G. Tian , G. A. Barragan , L. Guo , C. M. Amador , N. Ghatwai , P. Rathi , M. S. Wood , Y. Li , C. Zhang , T. Demberg , E. J. Di Pierro , A. C. Sher , H. Zhang , B. Mehta , S. G. Thakkar , B. Grilley , T. Wang , B. D. Weiss , A. Montalbano , M. Subramaniam , C. Xu , C. Sachar , D. K. Wells , G. Dotti , L. S. Metelitsa , Nat. Med. 2023, 29, 1379. 37188782 10.1038/s41591-023-02363-y

[9] X. Zhou , Y. Wang , Z. Dou , G. Delfanti , O. Tsahouridis , C. M. Pellegry , M. Zingarelli , G. Atassi , M. G. Woodcock , G. Casorati , P. Dellabona , W. Y. Kim , L. Guo , B. Savoldo , A. Tsagaratou , J. J. Milner , L. S. Metelitsa , G. Dotti , Nat Cancer 2024, 5, 1607. 39354225 10.1038/s43018-024-00830-0 PMC12002392

[10] P. Zhang , G. Zhang , X. Wan , J. Hematol. Oncol. 2023, 16, 97. 37596653 10.1186/s13045-023-01492-8 PMC10439661

[11] J. Radler , D. Gupta , A. Zickler , S. E. Andaloussi , Mol. Ther. 2023, 31, 1231. 36805147 10.1016/j.ymthe.2023.02.013 PMC10188647

[12] G. van Niel , G. D'Angelo , G. Raposo , Nat. Rev. Mol. Cell Biol. 2018, 19, 213. 29339798 10.1038/nrm.2017.125

[13] P. Xia , H. Yuan , M. Tian , T. Zhong , R. Hou , X. Xu , J. Ma , H. Wang , Z. Li , D. Huang , C. Qu , L. Dai , C. Xu , C. Yang , H. Jiang , Y. He , F. Rückert , Z. Li , Y. Yuan , J. Wang , Adv. Funct. Mater. 2023, 33, 2209393.

[14] L. Rao , Y. Yuan , X. Shen , G. Yu , X. Chen , Nat. Nanotechnol. 2024, 19, 1769. 39362960 10.1038/s41565-024-01753-8

[15] Q.‐F. Meng , Y. Han , Y. Liu , P. Pan , R.‐C. Chen , H. Zhang , L. Rao , Cell Biomater. 2025, 1, 100017.

[16] X. Lin , L. Yue , K. Cheng , L. Rao , Acc. Mater. Res. 2025, 6, 327.

[17] D. Li , R. Wang , T. Liang , H. Ren , C. Park , C. H. Tai , W. Ni , J. Zhou , S. Mackay , E. Edmondson , J. Khan , B. S. Croix , M. Ho , Nat. Commun. 2023, 14, 5920. 37739951 10.1038/s41467-023-41631-w PMC10517151

[18] W. Fu , C. Lei , S. Liu , Y. Cui , C. Wang , K. Qian , T. Li , Y. Shen , X. Fan , F. Lin , M. Ding , M. Pan , X. Ye , Y. Yang , S. Hu , Nat. Commun. 2019, 10, 4355. 31554797 10.1038/s41467-019-12321-3 PMC6761190

[19] G. Chen , X. She , Y. Yin , J. Ma , Y. Gao , H. Gao , H. Qin , J. Fang , Signal Transduct. Target Ther. 2022, 7, 350. 36229443 10.1038/s41392-022-01177-7 PMC9561108

[20] C. I. Lin , A. Merley , T. E. Sciuto , D. Li , A. M. Dvorak , J. M. Melero‐Martin , H. F. Dvorak , S. C. Jaminet , Angiogenesis 2014, 17, 897. 24986520 10.1007/s10456-014-9437-2 PMC4177288

[21] D. C. Seo , J. M. Sung , H. J. Cho , H. Yi , K. H. Seo , I. S. Choi , D. K. Kim , J. S. Kim , A. A. El‐Aty , H. C. Shin , Mol. Cancer 2007, 6, 75. 18034892 10.1186/1476-4598-6-75 PMC2234429

[22] S. Sun , Z. Ding , X. Yang , X. Zhao , M. Zhao , L. Gao , Q. Chen , S. Xie , A. Liu , S. Yin , Z. Xu , X. Lu , Int. J. Nanomed. 2021, 16, 2337. 10.2147/IJN.S297631 PMC7997558 33790553

[23] C. Bao , Q. Gao , L. L. Li , L. Han , B. Zhang , Y. Ding , Z. Song , R. Zhang , J. Zhang , X. H. Wu , Biomolecules 2021, 11, 1020238. 10.3390/biom11020238 PMC7914546 33567640

[24] S. Sun , Z. Ding , L. Gao , B. D. Hammock , X. Huang , Z. P. Xu , X. Wang , Q. Cheng , F. Mo , W. Shi , S. Xie , A. Liu , H. Li , X. Yang , X. Lu , Theranostics 2023, 13, 5099. 37771772 10.7150/thno.84946 PMC10526666

[25] Q. Tang , J. Chen , Z. Di , W. Yuan , Z. Zhou , Z. Liu , S. Han , Y. Liu , G. Ying , X. Shu , M. Di , J. Exp. Clin. Cancer Res. 2020, 39, 232. 33153498 10.1186/s13046-020-01690-z PMC7643364

[26] Y. K. Huang , X. G. Fan , F. Qiu , Int. J. Mol. Sci. 2016, 17, 661. 27153056 10.3390/ijms17050661 PMC4881487

[27] J. Cao , J. C. Yang , V. Ramachandran , T. Arumugam , D. F. Deng , Z. S. Li , L. M. Xu , C. D. Logsdon , Cell. Physiol. Biochem. 2016, 39, 740. 27459514 10.1159/000445664

[28] C. Zhu , X. Luo , J. Wu , Y. Liu , L. Liu , S. Ma , R. Xie , S. Wang , W. Ji , J. Cell. Mol. Med. 2021, 25, 2356. 31876386 10.1111/jcmm.14787 PMC7933925

[29] Y. R. Kao , J. Y. Shih , W. C. Wen , Y. P. Ko , B. M. Chen , Y. L. Chan , Y. W. Chu , P. C. Yang , C. W. Wu , S. R. Roffler , Clin. Cancer Res. 2003, 9, 2807. 12855661

[30] T. E. Sciuto , A. Merley , C. I. Lin , D. Richardson , Y. Liu , D. Li , A. M. Dvorak , H. F. Dvorak , S. C. Jaminet , Biochem. Biophys. Res. Commun. 2015, 465, 338. 26241677 10.1016/j.bbrc.2015.07.142 PMC4579096

[31] Y. Shen , G. Liu , Q. Zhang , X. Tian , L. Ouyang , L. Zhang , Immunol. Lett. 2023, 255, 1. 36739093 10.1016/j.imlet.2023.01.011

[32] A. Visintin , K. Knowlton , E. Tyminski , C. I. Lin , X. Zheng , K. Marquette , S. Jain , L. Tchistiakova , D. Li , C. J. O'Donnell , A. Maderna , X. Cao , R. Dunn , W. B. Snyder , A. K. Abraham , M. Leal , S. Shetty , A. Barry , L. Zawel , A. J. Coyle , H. F. Dvorak , S. C. Jaminet , Mol. Cancer Ther. 2015, 14, 1868. 26089370 10.1158/1535-7163.MCT-15-0188

[33] R. C. Sterner , R. M. Sterner , Blood Cancer J. 2021, 11, 69. 33824268 10.1038/s41408-021-00459-7 PMC8024391

[34] B. Yang , Y. Chen , J. Shi , Adv. Mater. 2019, 31, 1802896. 10.1002/adma.201802896 30126052

[35] P. J. Peters , J. Borst , V. Oorschot , M. Fukuda , O. Krahenbuhl , J. Tschopp , J. W. Slot , H. J. Geuze , J. Exp. Med. 1991, 173, 1099. 2022921 10.1084/jem.173.5.1099 PMC2118839

[36] C. Fusco , G. De Rosa , I. Spatocco , E. Vitiello , C. Procaccini , C. Frige , V. Pellegrini , R. La Grotta , R. Furlan , G. Matarese , F. Prattichizzo , P. de Candia , J. Extracell. Vesicles 2024, 13, 12433. 10.1002/jev2.12433 PMC11089593 38738585

[37] J. Chou , E. A. Egusa , S. Wang , M. L. Badura , F. Lee , A. P. Bidkar , J. Zhu , T. Shenoy , K. Trepka , T. M. Robinson , V. Steri , J. Huang , Y. Wang , E. J. Small , E. Chan , B. A. Stohr , A. Ashworth , B. Delafontaine , S. Rottey , K. S. Cooke , N. Hashemi Sadraei , B. Yu , M. Salvati , J. M. Bailis , F. Y. Feng , R. R. Flavell , R. Aggarwal , Cancer Res. 2023, 83, 301. 36351060 10.1158/0008-5472.CAN-22-1433 PMC10263373

[38] A. Ghai , A. Zheleznyak , M. Mixdorf , J. O'Neal , J. Ritchey , M. Rettig , J. DiPersio , M. Shokeen , S. Achilefu , Eur. J. Nucl. Med. Mol. Imaging 2021, 48, 1302. 33179150 10.1007/s00259-020-05097-y PMC8110592

[39] O. Kepp , L. Menger , E. Vacchelli , C. Locher , S. Adjemian , T. Yamazaki , I. Martins , A. Q. Sukkurwala , M. Michaud , L. Senovilla , L. Galluzzi , G. Kroemer , L. Zitvogel , Cytokine Growth Factor Rev. 2013, 24, 311. 23787159 10.1016/j.cytogfr.2013.05.001

[40] C. Qu , H. Yuan , M. Tian , X. Zhang , P. Xia , G. Shi , R. Hou , J. Li , H. Jiang , Z. Yang , T. Chen , Z. Li , J. Wang , Y. Yuan , ACS Nano 2024, 18, 4019. 38253029 10.1021/acsnano.3c07002

[41] W. Zhong , S. Qin , B. Zhu , M. Pu , F. Liu , L. Wang , G. Ye , Q. Yi , D. Yan , J. Bio. Chem. 2015, 290, 8876. 25596532 10.1074/jbc.M114.610188 PMC4423679

[42] A. Wesle , E. Moraes Ribeiro , R. Schairer , H. Keppeler , F. Korkmaz , P. Radszuweit , K. Bieber , C. Lengerke , D. Schneidawind , C. Schneidawind , Cytotherapy 2025, 27, 7. 39269404 10.1016/j.jcyt.2024.08.004

[43] S. A. Jones , B. J. Jenkins , Nat. Rev. Immunol. 2018, 18, 773. 30254251 10.1038/s41577-018-0066-7

[44] A. Morath , W. W. Schamel , J. Leukoc. Biol. 2020, 107, 1045. 31994778 10.1002/JLB.2MR1219-233R

[45] M. E. Birnbaum , R. Berry , Y. S. Hsiao , Z. Chen , M. A. Shingu‐Vazquez , X. Yu , D. Waghray , S. Fischer , J. McCluskey , J. Rossjohn , T. Walz , K. C. Garcia , Proc. Natl. Acad. Sci. 2014, 111, 17576. 25422432 10.1073/pnas.1420936111 PMC4267357

[46] Q. Guo , Y. Jin , X. Chen , X. Ye , X. Shen , M. Lin , C. Zeng , T. Zhou , J. Zhang , Signal Transduct. Target Ther. 2024, 9, 53. 38433280 10.1038/s41392-024-01757-9 PMC10910037

[47] H. Yu , L. Lin , Z. Zhang , H. Zhang , H. Hu , Signal Transduct. Target Ther. 2020, 5, 209. 32958760 10.1038/s41392-020-00312-6 PMC7506548

[48] R. Jan , G. E. Chaudhry , Adv. Pharm. Bull. 2019, 9, 205. 31380246 10.15171/apb.2019.024 PMC6664112

[49] R. M. Siegel , M. J. Lenardo , Curr. Protoc. Immunol. 2002. 10.1002/0471142735.im1109cs44 18432870

[50] J. A. Ledbetter , W. E. Seaman , T. T. Tsu , L. A. Herzenberg , J. Exp. Med. 1981, 153, 1503. 6166718 10.1084/jem.153.6.1503 PMC2186177

[51] S. Ding , Y. Wang , Z. Liu , Y. Du , Y. Zhou , Y. Liu , J. Sun , Y. Li , L. Zeng , Regen. Ther. 2023, 24, 54. 37868719 10.1016/j.reth.2023.05.001 PMC10584668

[52] Y. Hu , W. Xu , H. Zeng , Z. He , X. Lu , D. Zuo , G. Qin , W. Chen , Br. J. Cancer 2020, 123, 1644. 32934344 10.1038/s41416-020-01040-y PMC7686370

[53] A. U. H. Khan , N. Allende‐Vega , D. Gitenay , S. Gerbal‐Chaloin , C. Gondeau , D. N. Vo , S. Belkahla , S. Orecchioni , G. Talarico , F. Bertolini , M. Bozic , J. M. Valdivielso , F. Bejjani , I. Jariel , I. C. Lopez‐Mejia , L. Fajas , C. H. Lecellier , J. Hernandez , M. Daujat , M. Villalba , Sci. Rep. 2017, 7, 10654. 28878225 10.1038/s41598-017-10339-5 PMC5587676

[54] P. Magati , R. Lencucha , Q. Li , J. Drope , R. Labonte , A. B. Appau , D. Makoka , F. Goma , R. Zulu , Tob. Control 2019, 28, 268. 29967193 10.1136/tobaccocontrol-2017-054213 PMC6512316

[55] D. C. Semenza , J. Interpers. Violence 2021, 36, 9255. 31370739 10.1177/0886260519864358

[56] J. M. Gilligan , Risk Anal 2021, 41, 1171. 31546286 10.1111/risa.13407 PMC8359220

[57] K. Okla , D. L. Farber , W. Zou , J. Exp. Med. 2021, 218, 20201605. 10.1084/jem.20201605 PMC7992502 33755718

[58] J. B. Williams , T. S. Kupper , Adv. Exp. Med. Biol. 2020, 39, 1273. 10.1007/978-3-030-49270-0_3 33119875

[59] B. J. Laidlaw , J. E. Craft , S. M. Kaech , Nat. Rev. Immunol. 2016, 16, 102. 26781939 10.1038/nri.2015.10 PMC4860014

