# Supplementary material for: Engineered CAR‐NKT Extracellular Vesicles Suppress Tumor Progression and Enhance Antitumor Immunity
Source: Adv Sci (Weinh). 2026 Jan 20;13(13):e21623. doi: 10.1002/advs.202521623 (PMC12955876; doi:10.1002/advs.202521623)
Supplement: Supplementary file 1 — Supporting Information [file ADVS-13-e21623-s001.docx]

**Engineered CAR-NKT Extracellular Vesicles Suppress Tumor Progression and Enhance Antitumor Immunity**

Xiaopei Hao^1,5^, Chengming Qu^2,4^, Yanzhao Zhou^3^, Xiaoqian Wang^1^, Xiangjun Qian^1^, Xun Chen^1^, Feng Han^1^, Xiaokai Zhang^1^, Yiyi Ji^2,6^, Han Li^2^, ChengWei Ju^2^, Peng Xia^2,4*^, Weiwei Tang^5*^, Hao Zhuang^1*^, Jinxue Zhou^1*^

^1^Department of Hepatobiliary and Pancreatic Surgery, The Affiliated Cancer Hospital of Zhengzhou University & Henan Cancer Hospital, Zhengzhou, China.

^2^Department of Chemistry, Department of Biochemistry and Molecular Biology, The University of Chicago, Chicago, IL, USA.

^3^Department of Medical Oncology, The Affiliated Cancer Hospital of Zhengzhou University & Henan Cancer Hospital, Zhengzhou, China.

^4^Zhongnan Hospital of Wuhan University, TaiKang Center for Life and Medical Sciences, Clinical Medicine Research Center for Minimally Invasive Procedure of Hepatobiliary & Pancreatic Diseases of Hubei Province, Wuhan University, Wuhan, China.

^5^Hepatobiliary Center, The First Affiliated Hospital of Nanjing Medical University; Key Laboratory of Liver Transplantation, Chinese Academy of Medical Sciences; NHC Key laboratory of Hepatobiliary cancers, Nanjing, China.

^6^Department of Biomedical Engineering, Duke University, Durham, North Carolina, USA.

*Corresponding authors:

Peng Xia, E-mail: [pengxia@uchicago.edu](mailto:pengxia@uchicago.edu)

Weiwei Tang, E-mail: tangweiwei7449@njmu.edu.cn

Hao Zhuang, E-mail: zlyyzhuanghao3011@zzu.edu.cn

Jinxue Zhou, email: zlyyzhoujx1143@zzu.edu.cn

**Supplementary Figures**


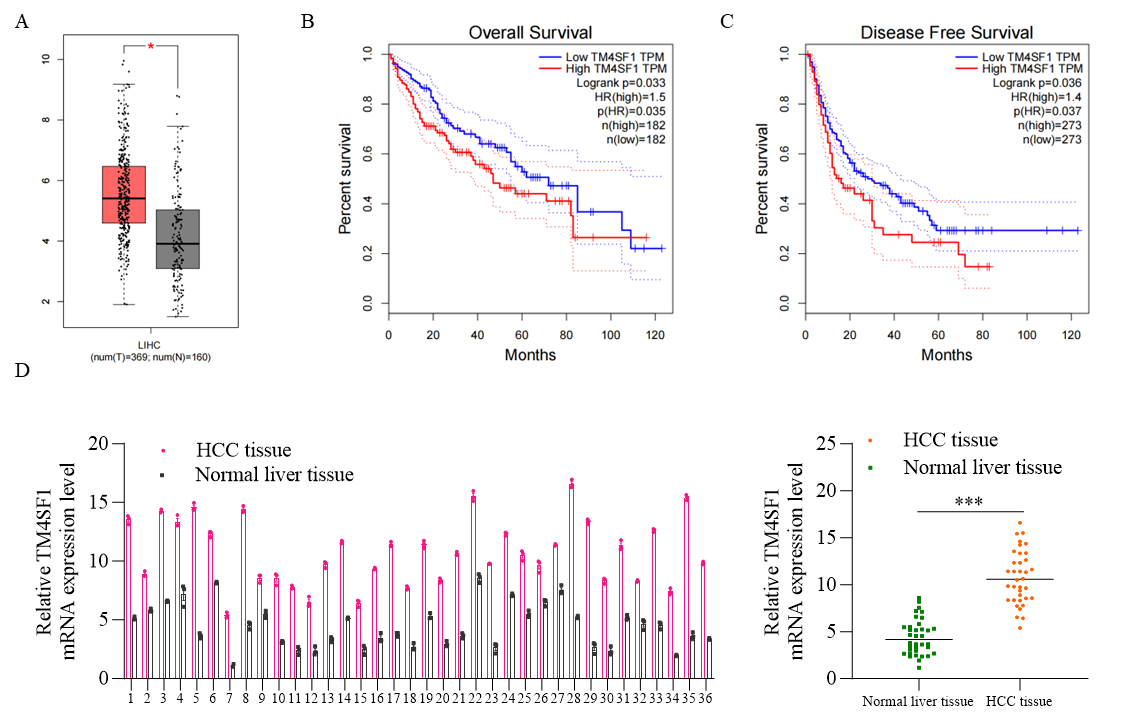


**Figure S1. High expression of TM4SF1 in HCC is significantly associated with poor prognosis.** (A) Bioinformatic analysis using the GEPIA database revealed that TM4SF1 expression was markedly elevated in 369 HCC tissues compared to 160 normal liver tissues. (B) High TM4SF1 expression was significantly correlated with reduced overall survival in HCC patients. (C) High TM4SF1 expression was significantly correlated with reduced disease-free survival in HCC patients. (D) qRT-PCR analysis further confirmed that TM4SF1 mRNA levels were significantly higher in 36 HCC tissues compared to their paired adjacent normal liver tissues. Quantitative data are presented as mean ± SD. Statistical significance was determined by unpaired t-test (A) and paired t-test (D), or Mantel–Cox test (B, C). Statistical significance indicated as ns (not significant), * *p* < 0.05, ** *p* < 0.01, *** *p* < 0.001.


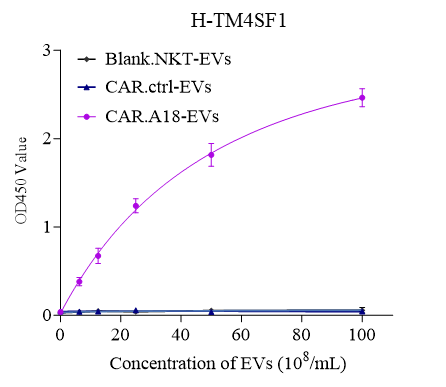


**Figure S2. Binding affinity of CAR^TM4SF1^-EVs to human TM4SF1 protein.** ELISA was performed to assess the binding activity of Blank.NKT-EVs, CAR.ctrl-EVs, and CAR.A18-EVs to human TM4SF1 protein (*n* = 3). The results demonstrated that CAR.A18-EVs exhibited significantly higher binding affinity compared to Blank.NKT-EVs and CAR.ctrl-EVs.


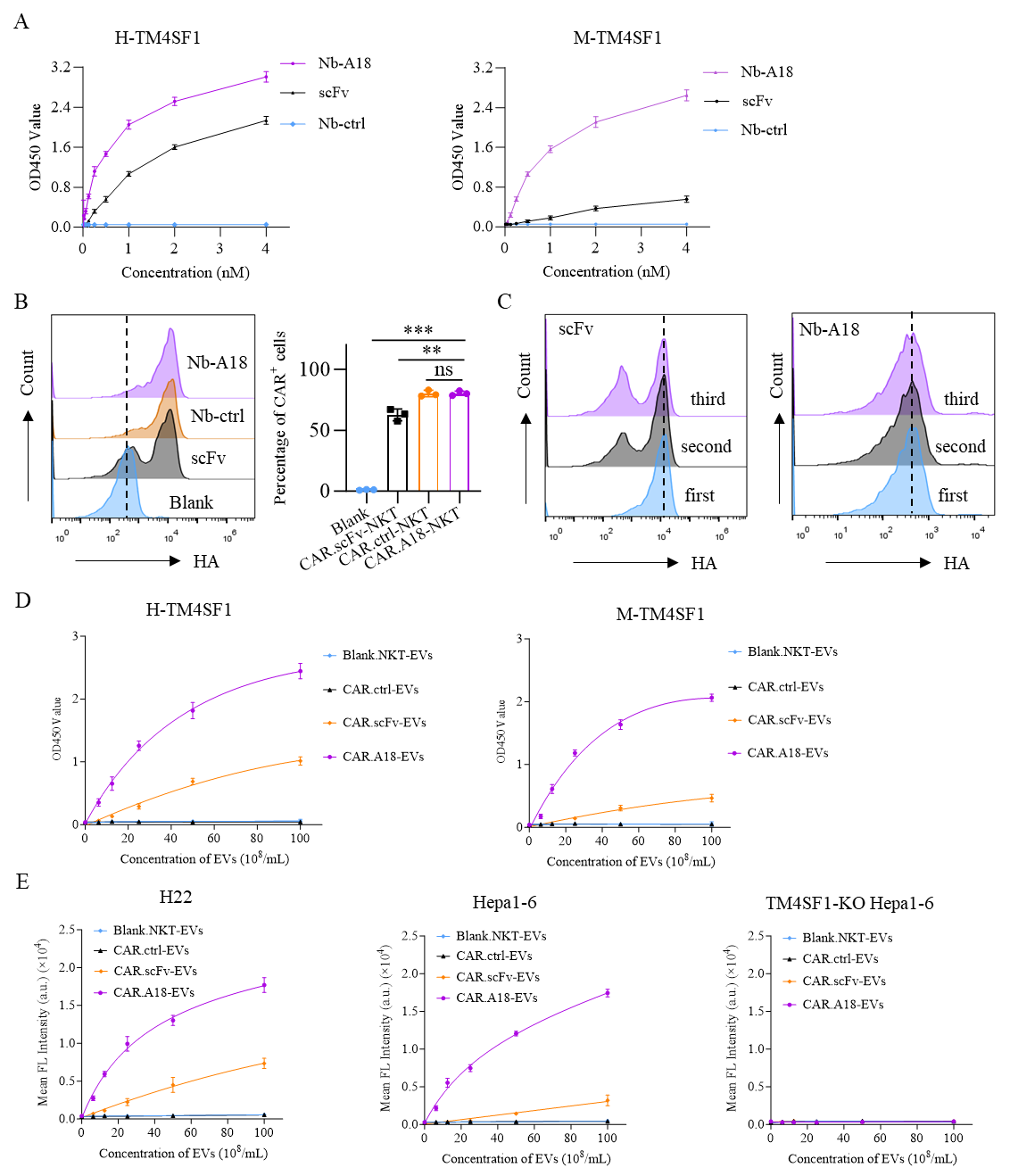


**Figure S3. Comparative analysis of CAR constructs based on TM4SF1 nanobody and scFv fragments.** (A) ELISA analysis of the binding affinity of Nb-A18 and scFv fragments to human and murine TM4SF1 proteins (*n* = 3). (B) Flow cytometric analysis of surface expression levels of different CAR constructs on NKT cells (*n* = 3). (C) Comparison of the functional persistence of NKT cells expressing different CAR constructs after multiple rounds of antigen stimulation. (D) ELISA assay evaluating the binding of EVs derived from different sources to human and murine TM4SF1 proteins (*n* = 3). (E) Cell-based ELISA showing the binding levels of EVs from various sources to H22 and Hepa1-6 cells, as well as TM4SF1-knockout Hepa1-6 cells (*n* = 3). Quantitative data are presented as mean ± SD. Statistical significance was calculated using one-way ANOVA followed by Tukey’s multiple comparisons test for multi-group analyses (B). Statistical significance is indicated as ns (not significant), * p < 0.05, ** p < 0.01, *** p < 0.001.


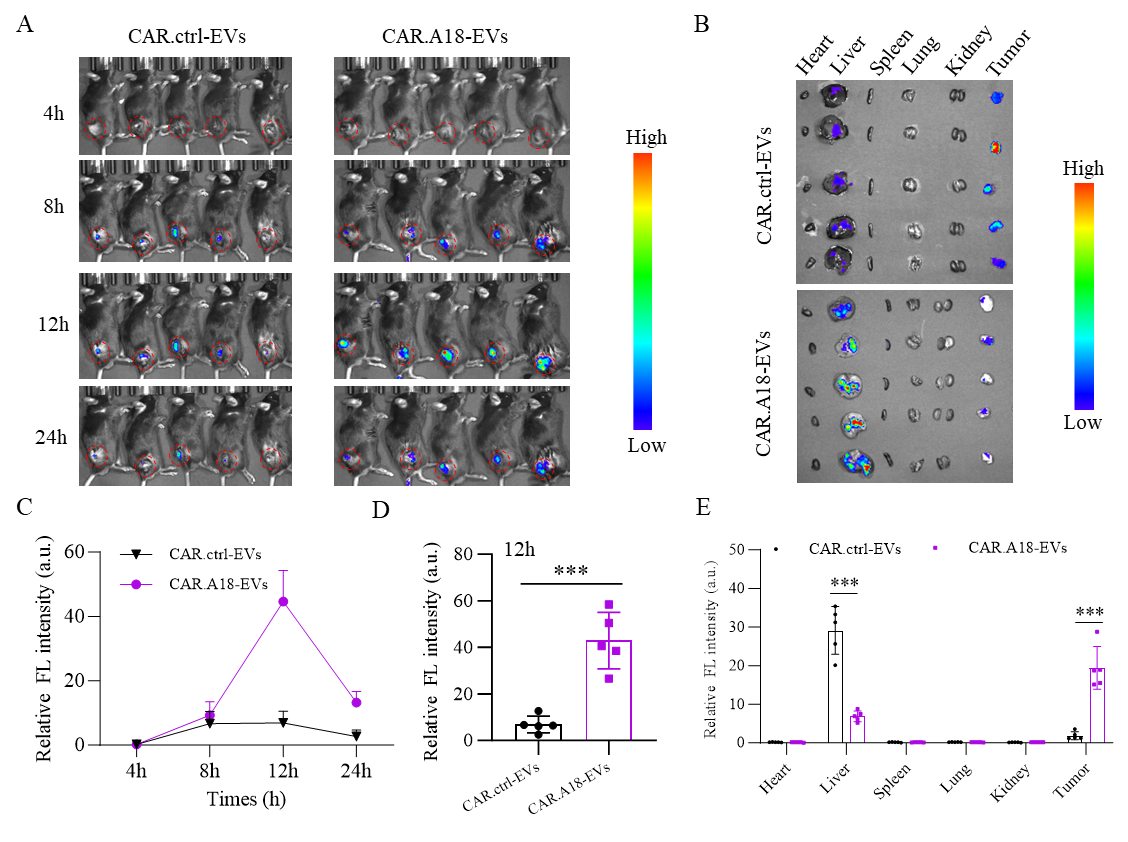


**Figure S4. Immunoaffinity-purified CAR.A18^+^ -EVs exhibit enhanced tumor targeting and markedly reduced nonspecific hepatic accumulation.** (A) In vivo fluorescence imaging of DiR-labeled CAR.ctrl-EVs and CAR.A18-EVs in Hepa1-6 tumor-bearing mice at different time points. (B) Ex vivo fluorescence imaging of tumors and major organs at 24 h post-injection. (C) Quantitative analysis of tumor fluorescence intensity over time from panel (A). The results showed that CAR.A18-EVs exhibited significantly increased fluorescence signals in tumors at 12 h, followed by a gradual decline (*n* = 5). (D) Statistical analysis of fluorescence intensity in the tumor region from NIR-I imaging at 12 h (panel A) (*n* = 5). (E) Quantitative analysis of ex vivo organ and tumor fluorescence from panel (B). CAR.A18-EVs displayed higher accumulation in tumor tissues compared with the heart, liver, lung, kidney, and spleen, whereas CAR.ctrl-EVs showed predominant nonspecific accumulation in the liver (*n* = 5). Quantitative data are presented as mean ± SD. Statistical significance was calculated using two-way ANOVA (C, E) or one-way ANOVA with Tukey’s post-test (D). Statistical significance is indicated as ns (not significant), * p < 0.05, ** p < 0.01, *** p < 0.001.


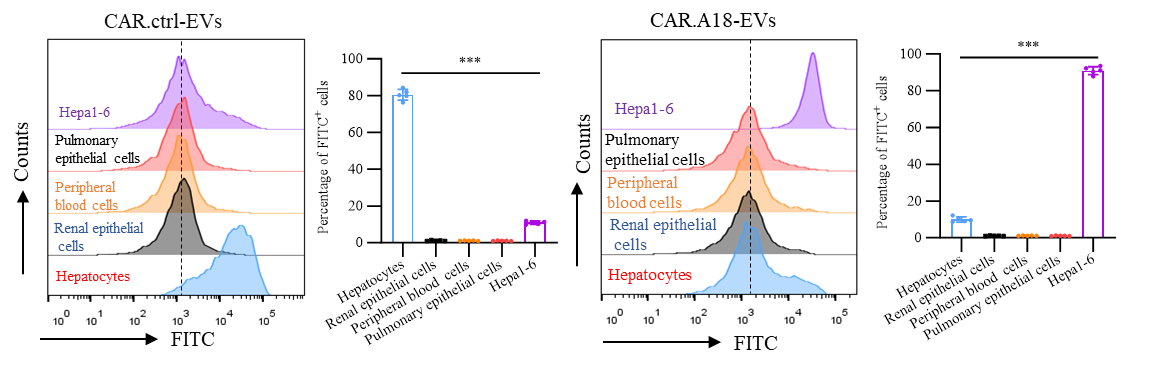


**Figure S5. Tumor-specific uptake and low nonspecific tissue distribution of CAR.A18-EVs.** Flow cytometry was used to assess the uptake of CAR.ctrl-EVs and CAR.A18-EVs by different cell types (*n* = 5). The results showed that CAR.ctrl-EVs exhibited weak uptake in TM4SF1-high Hepa1-6 tumor cells but showed relatively high nonspecific uptake in hepatocytes. In contrast, CAR.A18-EVs displayed markedly enhanced uptake in Hepa1-6 tumor cells, while uptake by renal epithelial cells, pulmonary epithelial cells, peripheral blood cells, and hepatocytes remained minimal, with no evident accumulation observed. Quantitative data are presented as mean ± SD. Statistical significance was calculated using one-way ANOVA with Tukey’s post-test. Statistical significance is indicated as ns (not significant), * p < 0.05, ** p < 0.01, *** p < 0.001.


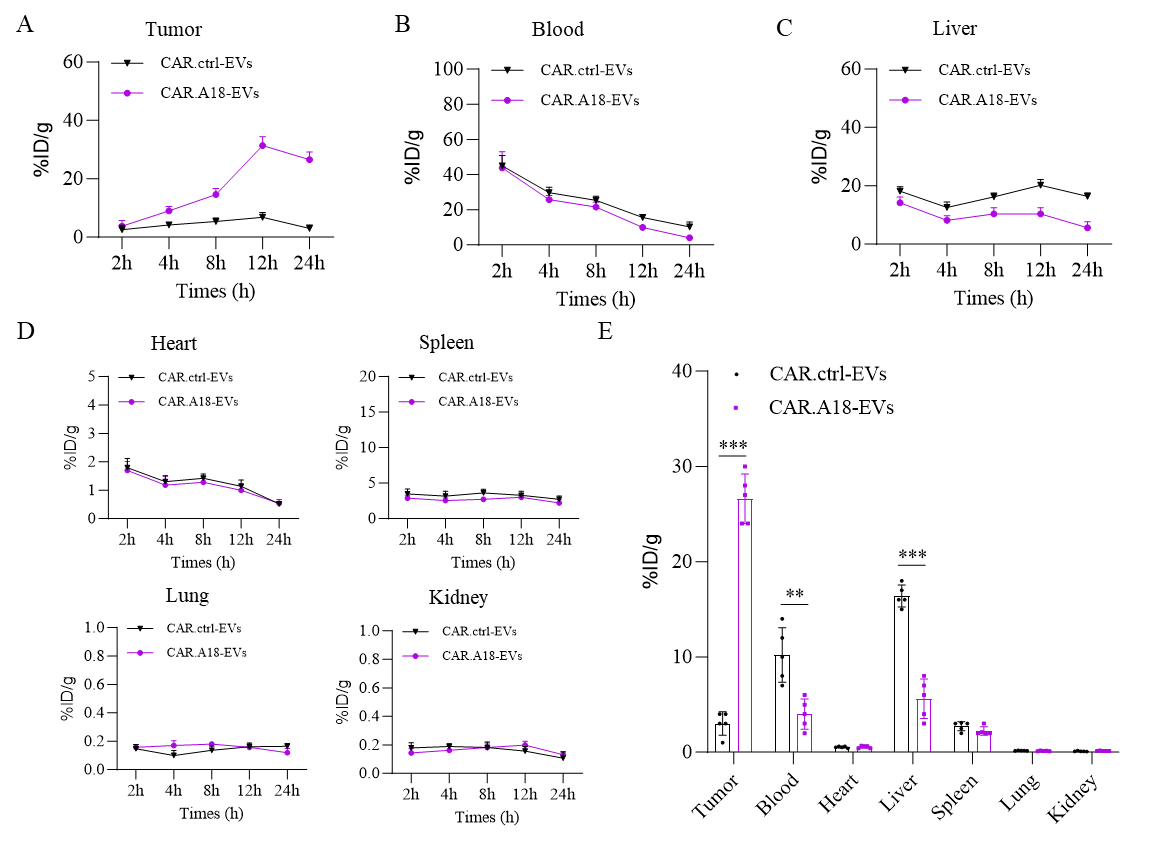


**Figure S6. PET quantification based on ⁸⁹Zr labeling reveals the pharmacokinetics and biodistribution profile of CAR.A18-EVs *in vivo*.** (A) ⁸⁹Zr-labeled CAR.A18-EVs or CAR.ctrl-EVs were intravenously injected into Hepa1-6 tumor-bearing mice, followed by PET imaging at 2 h, 4 h, 8 h, 12 h, and 24 h. Radioactive signals in tumor regions were quantitatively analyzed (*n* = 5). (B) Time-dependent changes of ⁸⁹Zr signals in the blood were monitored and quantified to evaluate the circulation clearance characteristics of the two EV groups (*n* = 5). (C) Time-course quantification of ⁸⁹Zr radioactivity in the liver was performed to compare the hepatic distribution dynamics of CAR.A18-EVs and CAR.ctrl-EVs (*n* = 5). (D) Quantitative analysis of ⁸⁹Zr radioactivity in major non-target organs (heart, spleen, lung, and kidney) was conducted to assess the biodistribution of the two EV groups in non-tumor tissues (*n* = 5). (E) At 24 h post-injection, the radioactivity in blood, tumor, and major organs was measured by γ-counting, and % ID/g values were calculated to characterize the in vivo distribution patterns of CAR.A18-EVs and CAR.ctrl-EVs (*n* = 5). Quantitative data are presented as mean ± SD. Statistical significance was calculated using two-way ANOVA (E). Statistical significance is indicated as ns (not significant), * p < 0.05, ** p < 0.01, *** p < 0.001.


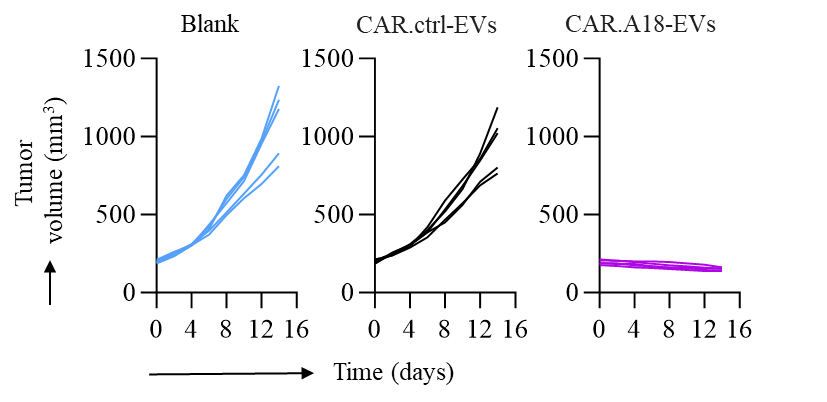


**Figure S7. Antitumor effects of CAR^TM4SF1^-EVs *in vivo*.** Hepa1-6 tumor–bearing mice were intravenously treated with PBS, CAR.ctrl-EVs, or CAR.A18-EVs. Tumor growth curves for individual mice in each group are shown (n = 5).


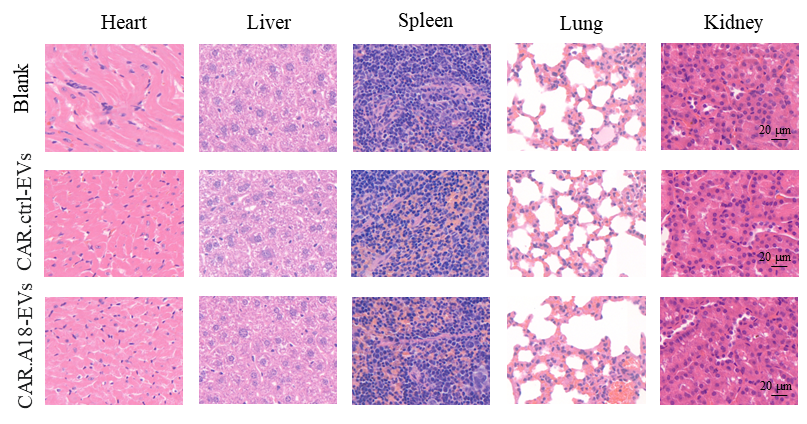


**Figure S8. *In vivo* safety analysis of CAR^TM4SF1^-EVs.** Hepa1-6 tumor-bearing mice were treated with PBS, CAR.ctrl-EVs, or CAR.A18-EVs via intravenous injection. H&E staining of major organs, including the heart, liver, spleen, lungs, and kidneys, was performed to assess potential histopathological changes in all groups.


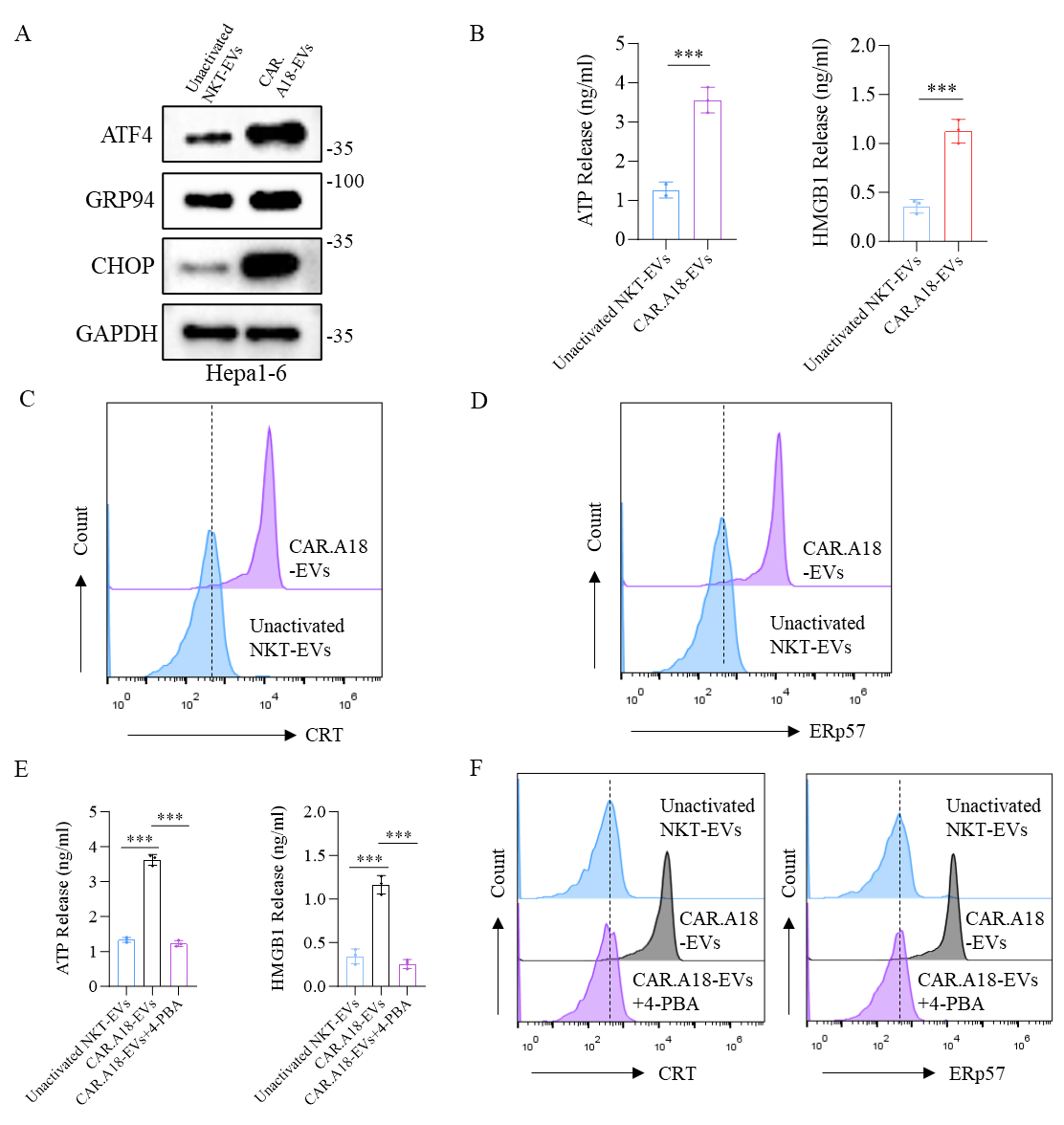


**Figure S9. CAR.A18-EVs induce ICD in tumor cells through ER stress.** (A) Western blot analysis showing the expression levels of ER stress–related proteins ATF4, GRP94, and CHOP in tumor cells treated with CAR.A18-EVs. (B) ELISA quantification of extracellular ATP and HMGB1 release following CAR.A18-EV treatment (*n* = 3). (C) Flow cytometry analysis of surface CRT exposure on tumor cells. (D) Flow cytometry analysis of surface ERp57 exposure on tumor cells. (E) After inhibition of ER stress with 4-PBA (4 mM), ELISA measurement of extracellular ATP and HMGB1 release (*n* = 3). (F) Following 4-PBA (4 mM) treatment, flow cytometry analysis of surface CRT and ERp57 exposure. Quantitative data are presented as mean ± SD. Statistical significance was calculated one-way ANOVA with Tukey’s post-test (B, E). Statistical significance is indicated as ns (not significant), * p < 0.05, ** p < 0.01, *** p < 0.001.


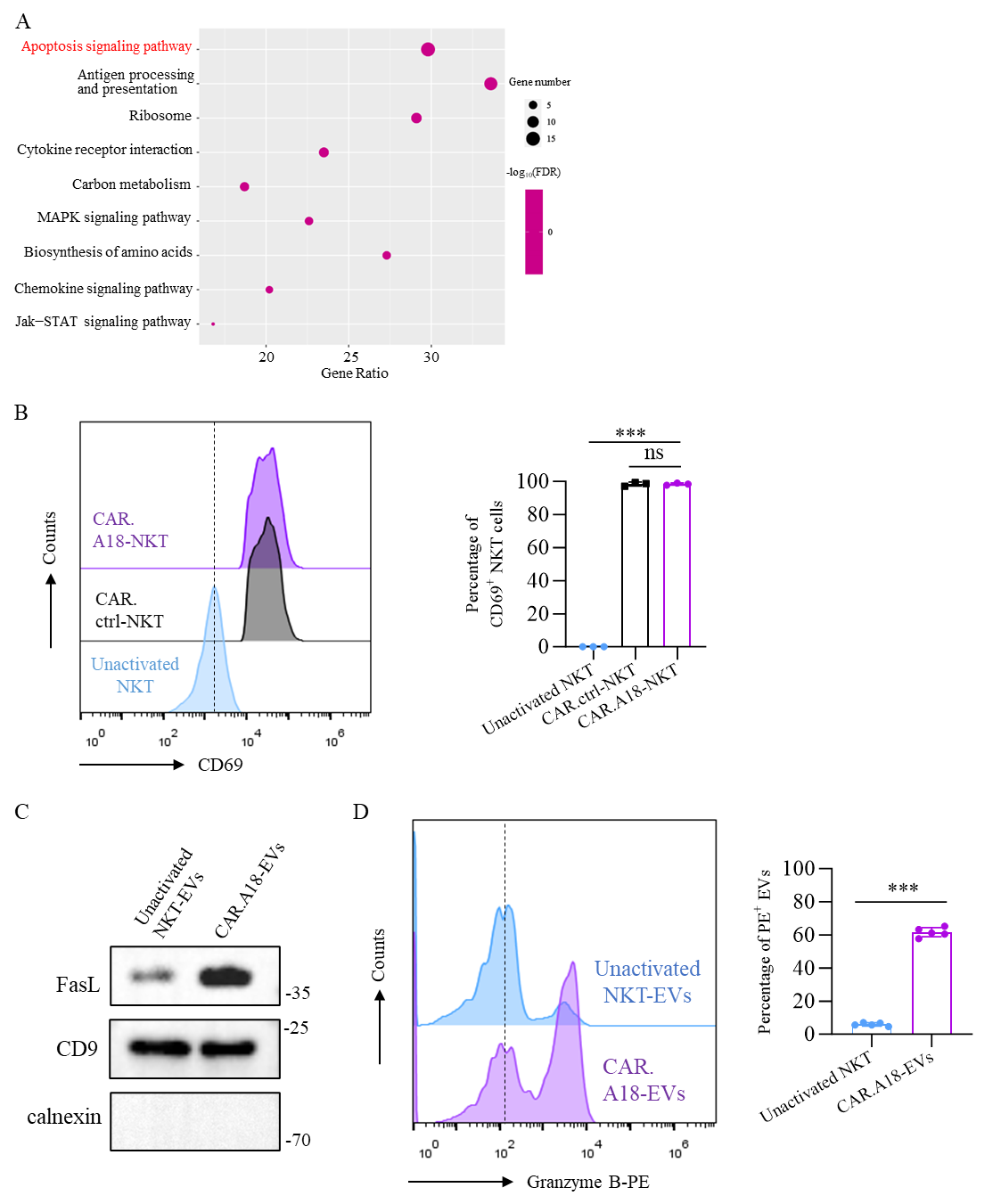


**Figure S10. Activation of CAR-NKT Cells Enhances Functional Protein Loading of CAR.A18-EVs.** (A) KEGG pathway enrichment analysis based on quantitative proteomics shows that CAR.A18-EVs cargo is significantly enriched in apoptosis, immune cytokine, and chemokine signaling pathways. (B) Flow cytometry analysis of CD69 expression in unactivated NKT cells, activated CAR.ctrl-NKT cells, and activated CAR.A18-NKT cells to assess the activation status of CAR-NKT cells (*n* = 3). (C) Western blot analysis of FasL, CD9, and calnexin protein expression in EVs derived from unactivated NKT cells and activated CAR.A18-NKT cells. (D) Nano-flow cytometry analysis of intracellular Granzyme B levels within EVs (*n* = 3). Quantitative data are presented as mean ± SD. Statistical significance was calculated using one-way ANOVA with Tukey’s post-test (B, D). Statistical significance is indicated as ns (not significant), * p < 0.05, ** p < 0.01, *** p < 0.001.


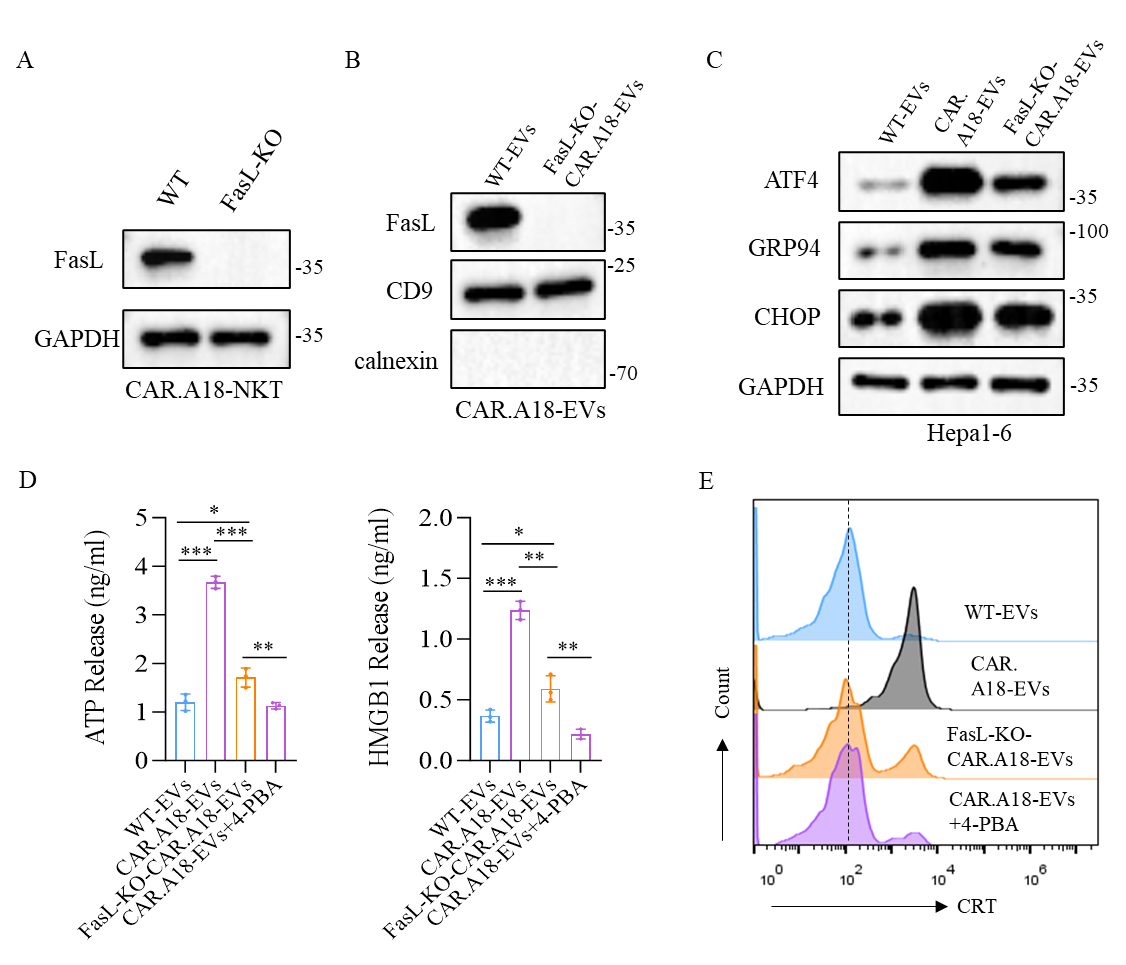


**Figure S11. FasL mediates CAR.A18-EVs–induced ICD through ER stress activation.** (A) Western blot verification confirms the absence of FasL protein expression in Faslg-knockout CAR.A18-NKT cells. (B) Western blot verification confirms the absence of FasL protein expression in EVs secreted by Faslg-knockout CAR.A18-NKT cells. (C) Western blot analysis of ER stress–related proteins ATF4, GRP94, and CHOP in tumor cells under different treatment conditions. (D) ELISA quantification of extracellular ATP and HMGB1 release shows that DAMPs release is significantly decreased after FasL knockout or 4-PBA (4 mM) treatment (*n* = 3). (E) Flow cytometry analysis that FasL deficiency or 4-PBA (4 mM) intervention significantly reduces CRT exposure induced by CAR.A18-EVs. Quantitative data are presented as mean ± SD. Statistical significance was calculated using two-tailed unpaired t-test analysis (D). Statistical significance is indicated as ns (not significant), * p < 0.05, ** p < 0.01, *** p < 0.001.


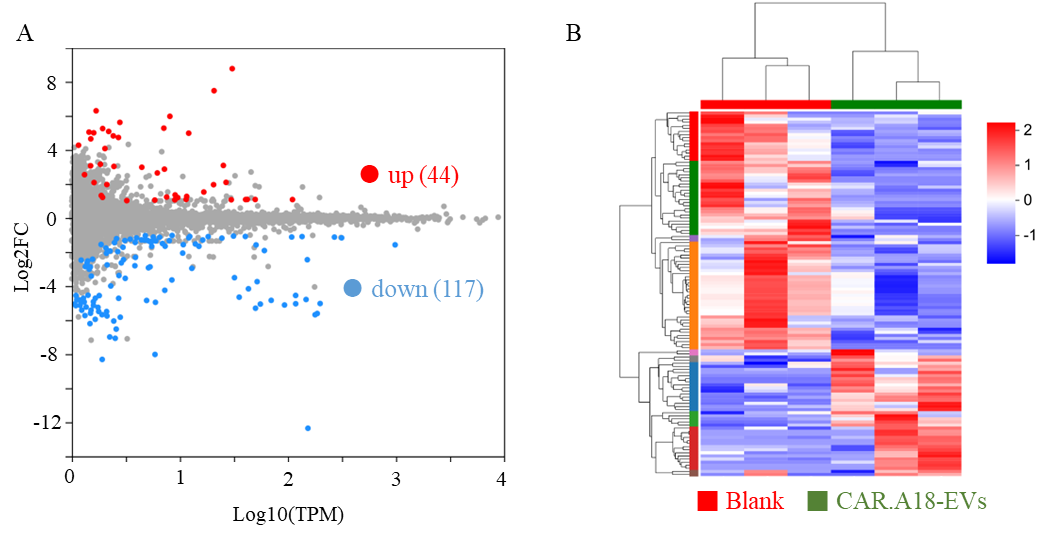


**Figure S12.** **Molecular mechanism analysis of the antitumor effects of CAR^TM4SF1^-EVs in vivo.** (A) RNA-seq analysis of differential gene expression in tumor tissues from the Blank and CAR.A18-EVs groups, with upregulated genes shown in red and downregulated genes in blue. (B) Heatmap illustrating the differences in gene expression between the Blank and CAR.A18-EVs groups, with the Blank group indicated in red and the CAR.A18-EVs group in green.


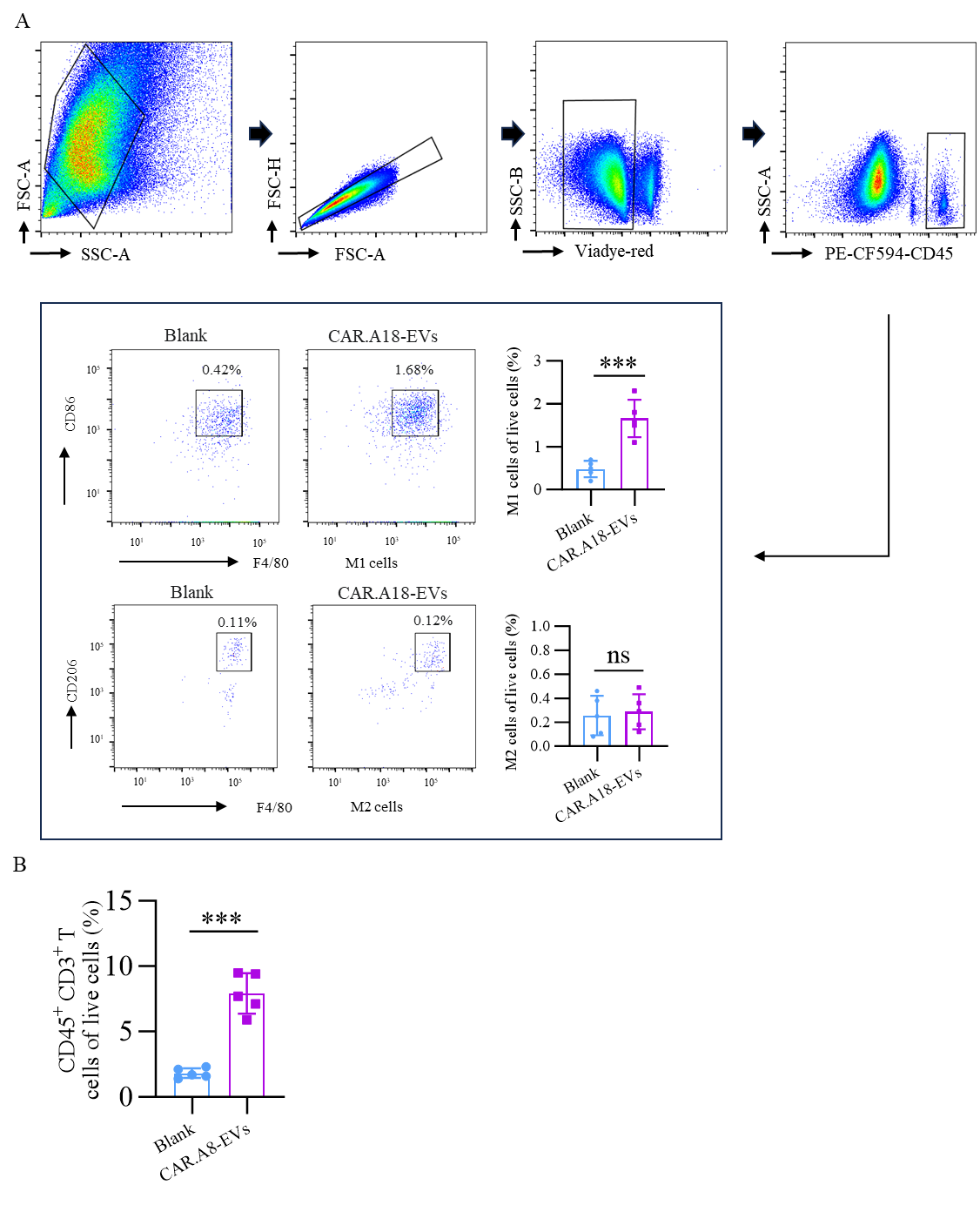


**Figure S13. Immunological mechanism analysis of the antitumor effects of CAR.TM4SF1-EVs *in vivo*.** (A) A comprehensive antibody labeling strategy and gating details were employed to analyze and quantify M1 macrophages (CD45⁺ F4/80⁺ CD86⁺) and M2 macrophages (CD45⁺ F4/80⁺ CD206⁺) in tumor tissues from the Blank group and the CAR.A18-EVs group using flow cytometry (n = 5). The results showed no significant differences in the number of M2 macrophages between the two groups. (B) Flow cytometry was used to analyze and quantify the number of CD45^+^ CD3^+^ T cells in tumor tissues from the Blank and CAR.A18-EVs groups (*n* = 5). The results demonstrated that the number of CD45^+^ CD3^+^ T cells was significantly higher in the CAR.A18-EVs group compared to the Blank group. Quantitative data are presented as mean ± SD. Statistical significance was calculated using one-way ANOVA with Tukey’s post-test. Statistical significance is indicated as ns (not significant), * p < 0.05, ** p < 0.01, *** p < 0.001.


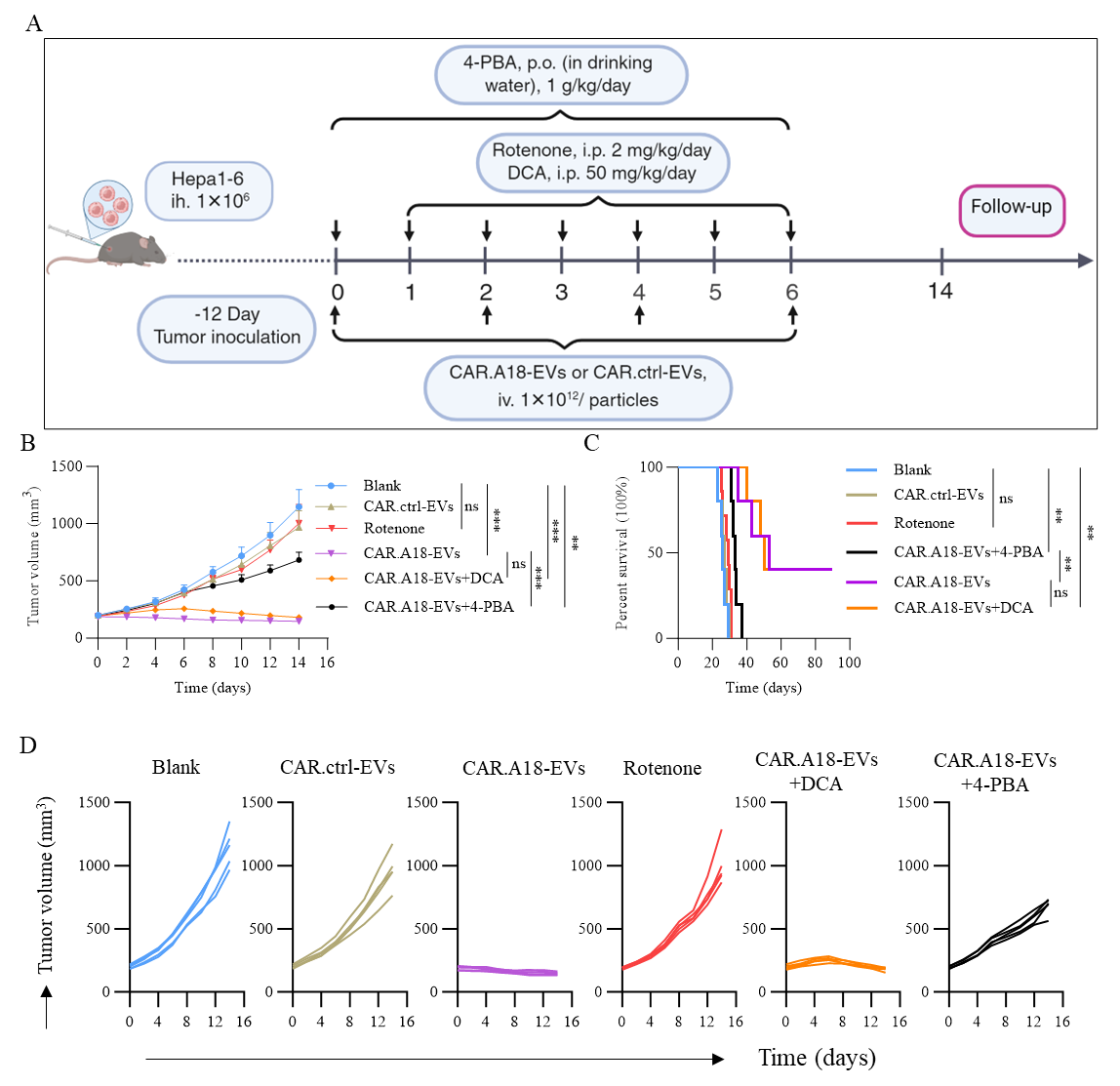


**Figure S14. Validation of the critical role of the ER stress pathway in the antitumor effects mediated by CAR.A18-EVs.** (A) Schematic illustration of the treatment schedule in the Hepa1-6 subcutaneous tumor model. The mice were randomly assigned into six groups: Blank (PBS, 200 µL); CAR.ctrl-EVs (1 × 10^12^ particles/injection); CAR.A18-EVs (1 × 10^12^ particles/injection); Rotenone (i.p. 2 mg/kg/day)); CAR.A18-EVs (1 × 10^12^ particles/injection) + DCA (i.p. 50 mg/kg/day)); CAR.A18-EVs (1 × 10^12^ particles/injection) + 4-PBA (p.o. 1 g/kg/day). (B) Tumor volumes were recorded and monitored under different treatment conditions *(n* = 5). (C) Survival curves of mice from each group were analyzed to evaluate the impact of different treatments on overall survival (*n* = 5). (D) Individual tumor growth curves of mice in each group were presented to illustrate variations in tumor progression among different treatments. Quantitative data are presented as mean ± SD. Statistical significance was calculated using two-way ANOVA with Tukey’s post-test (B) or Mantel–Cox test (C). Statistical significance is indicated as ns (not significant), * p < 0.05, ** p < 0.01, *** p < 0.001.


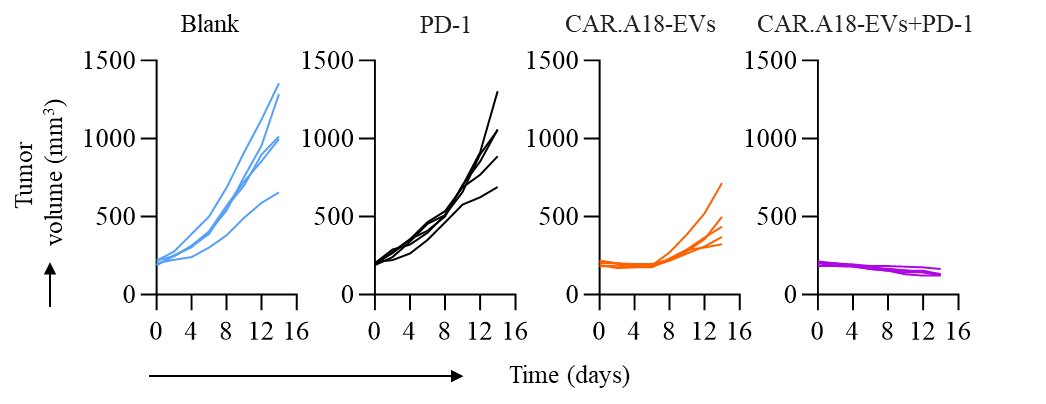


**Figure S15. *In vivo* antitumor effects of CAR.TM4SF1-EVs combined with PD-1 antibody.** Hepa1-6 tumor-bearing mice were treated with PBS, PD-1 antibody, CAR.A18-EVs, or CAR.A18-EVs plus PD-1 antibody via intravenous injection. Tumor volume changes for each mouse in the four groups are shown.

**Figure S16. Blood routine analysis of mice treated with CAR.TM4SF1-EVs combined with PD-1 antibody.** Hepa1-6 tumor-bearing mice were treated with PBS, PD-1 antibody, CAR.A18-EVs, or CAR.A18-EVs+PD-1 antibody via intravenous injection. Blood routine analysis was performed for all four groups of mice (*n* = 5). Quantitative data are presented as mean ± SD. Statistical significance was calculated using one-way ANOVA with Tukey’s post-test. Statistical significance is indicated as ns (not significant), * *p* < 0.05, ** *p* < 0.01, *** *p* < 0.001.


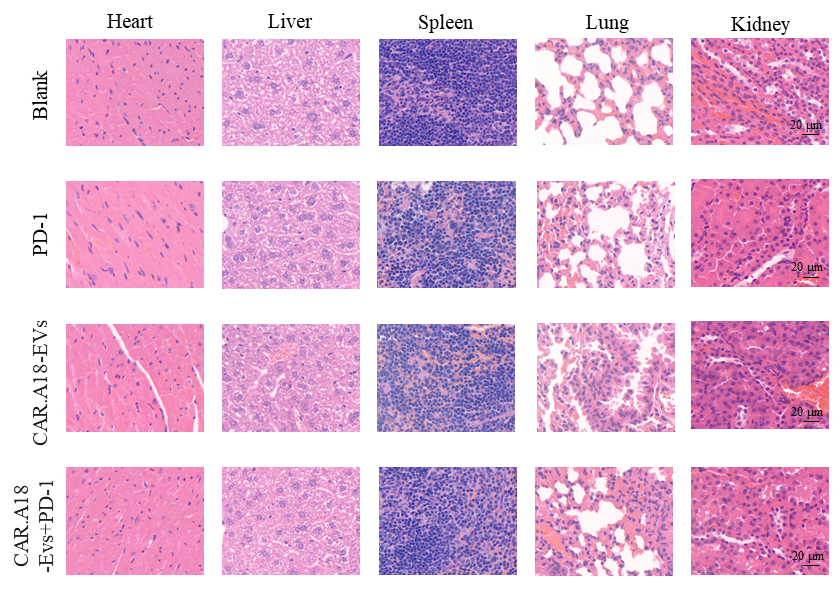


**Figure S17. H&E staining analysis of major organs in mice treated with CAR^TM4SF1^-EVs combined with PD-1 antibody.** Hepa1-6 tumor-bearing mice were treated with PBS, PD-1 antibody, CAR.A18-EVs, or CAR.A18-EVs plus PD-1 antibody via intravenous injection. H&E staining was performed on major organs, including the heart, liver, spleen, lungs, and kidneys, from all four groups of mice.


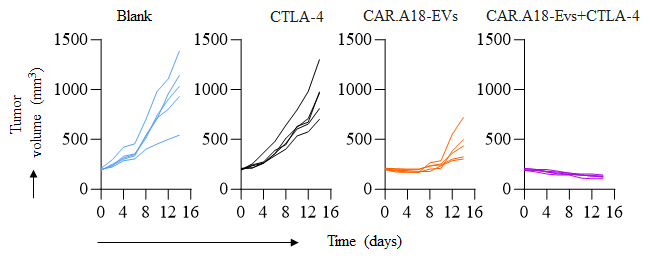


**Figure S18. *In vivo* antitumor effects of CAR^TM4SF1^-EVs combined with CTLA-4 antibody.** Hepa1-6 tumor-bearing mice were treated with PBS, CTLA-4 antibody, CAR.A18-EVs, or CAR.A18-EVs plus CTLA-4 antibody via intravenous injection. Tumor volume changes for each mouse in the four groups are shown.

**Figure S19. Blood routine analysis of mice treated with CAR.TM4SF1-EVs combined with CTLA-4 antibody.** Hepa1-6 tumor-bearing mice were treated with PBS, CTLA-4 antibody, CAR.A18-EVs, or CAR.A18-EVs+CTLA-4 antibody via injection. Blood routine analysis was performed for all four groups of mice (*n* = 5). Quantitative data are presented as mean ± SD. Statistical significance was calculated using one-way ANOVA with Tukey’s post-test. Statistical significance is indicated as ns (not significant), * *p* < 0.05, ** *p* < 0.01, *** *p* < 0.001.


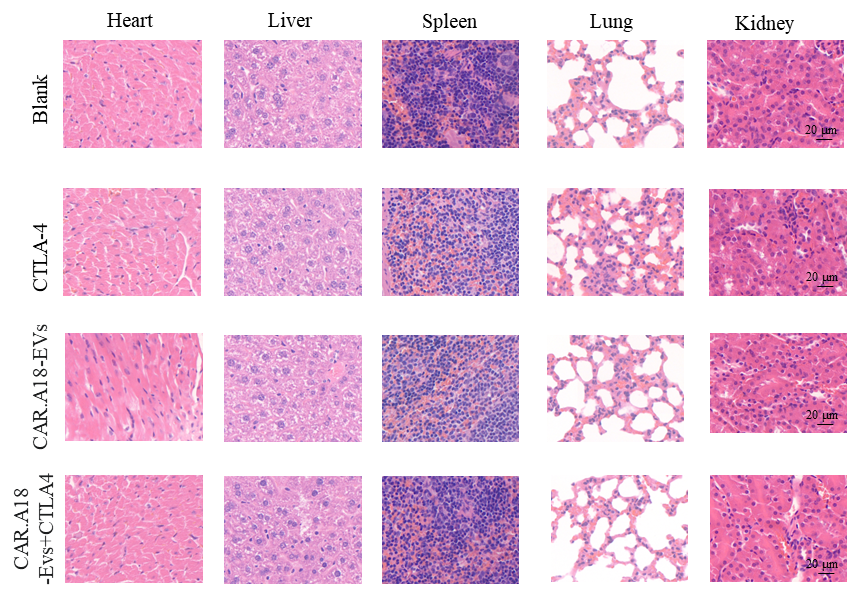


**Figure S20.** **H&E staining analysis of major organs in mice treated with CAR^TM4SF1^-EVs combined with CTLA-4 antibody.** Hepa1-6 tumor-bearing mice were treated with PBS, CTLA-4 antibody, CAR.A18-EVs, or CAR.A18-EVs plus CTLA-4 antibody via injection. H&E staining was performed on major organs, including the heart, liver, spleen, lungs, and kidneys, from all four groups of mice.

**Supplementary materials and methods**

**Experimental Reagents**

The cell culture reagents, including DMEM and RPMI-1640 media, fetal bovine serum (FBS), and penicillin-streptomycin (Pen-Strep), were sourced from Thermo Fisher Scientific (Waltham, MA, USA). The Annexin V-FITC/PI apoptosis detection kit was procured from BioLegend (San Diego, CA, USA), while 4% paraformaldehyde (PFA) was obtained from Boston BioProducts (Milford, MA, USA). Key experimental tools, including the PKH67 Green Fluorescent Cell Linker Mini Kit, TUNEL staining kit, and Bicinchoninic Acid (BCA) protein assay kit, were purchased from Sigma-Aldrich (St. Louis, MO, USA). Ultracentrifuge tubes were supplied by Beckman Coulter (Fullerton, CA, USA). The CCK-8 kit was obtained from Yeasen (Shanghai, China), and 2-(4-Amidinophenyl)-6-indolecarbamidine dihydrochloride (DAPI) was also sourced from Sigma-Aldrich. DCA, 4-PBA and Rotenone were purchased from MedChemExpress (MCE, NJ, USA). Details of the primary antibodies used are provided in **Supplementary Table S1**.

**Surface Plasmon Resonance**

SPR experiments were conducted using a Biacore S200 instrument (GE Healthcare) to evaluate the binding kinetics of nanobodies to human and mouse TM4SF1 proteins. All experiments were performed at 25°C in a buffer containing 10 mM HEPES (pH 7.4), 150 mM NaCl, 3 mM EDTA, and 0.05% Tween-20. Human and mouse TM4SF1 proteins were immobilized onto an S-CM5 series sensor chip (Cytiva) via amine coupling (NHS/EDC) to designated flow cells, with a mock-coupled surface used for background subtraction. Serial two-fold dilutions of Nbs, ranging from 3.9 nM to 62.5 nM, were injected over the sensor chip surface at a flow rate of 30 µL/min for 120 seconds, followed by a 180-second dissociation phase. The surface was regenerated after each cycle using 10 mM glycine (pH 2.0) for 120 seconds. Binding data were analyzed using the Biacore S200 evaluation software and fitted to a 1:1 interaction model based on the Langmuir binding equation.

**TM4SF1 Nanobody Library Screening**

Nanobodies targeting TM4SF1 were identified using a VHH phage display library (Shenzhen Kangtai Biotechnology) comprising approximately 2 × 10⁹ unique clones, as previously described. Panning was performed in immunotubes coated with human TM4SF1 protein. After three rounds of panning, 192 individual clones were selected for analysis using phage ELISA with horseradish peroxidase (HRP)-conjugated anti-M13 secondary antibody. The reaction was developed using TMB substrate solution, and the absorbance at 450 nm was measured within 30 minutes following the addition of the stop solution. Clones that demonstrated consistent positive results across all three rounds of phage ELISA were subsequently sequenced for further characterization.

**Plasmid Construction and Lentivirus Production**

The CAR retroviral vector used in this study has been previously described[1]. To construct the retroviral vector for CAR.TM4SF1, the scFv sequence of CAR.mB7-H3 was replaced with the sequence encoding the TM4SF1 nanobody. The preparation of retroviral supernatants for the transduction of mouse NKT cells followed established protocols. Briefly, HEK-293T cells were seeded in 10 cm culture dishes and transfected using GeneJuice transfection reagent with a mixture of retroviral vectors and pCL-Eco plasmids encoding gag, pol, and env. For lentiviral production, psPAX2 and pMD2G plasmids were used. The retroviral supernatants were collected at 48 and 72 hours post-transfection, then filtered through a 0.45 µm membrane filter.

**Extraction and Transduction of NKT Cells**Mouse NKT cells were isolated from spleens of C57BL/6J mice and activated on plates coated with anti-mouse CD3 monoclonal antibody (1 µg/mL) and anti-mouse CD28 monoclonal antibody (1 µg/mL). Cells were cultured in RPMI 1640 medium supplemented with mouse IL-2 (30 U/mL) for 48 hours[2]. Activated NKT cells were transduced with retroviral supernatants on retronectin-coated plates (Takara Bio) following the protocol previously established for human T cell transduction[3]. Post-transduction, cells were expanded in RPMI 1640 medium supplemented with mouse IL-2 (30 U/mL) and IL-7 (10 ng/mL), with medium refreshed every 2 days. On days 7–10, the expanded cells were harvested for use in functional assays conducted both in vitro and in vivo.

**Extracellular vesicles Isolation**

EVs were isolated from CAR.A18-NKT cells using a Beckman Optima XPN-100 ultracentrifuge. Briefly, CAR.A18-NKT cells were cultured for four days in medium supplemented with EV-depleted FBS to allow EV production. The collected culture supernatant was first centrifuged at 2,000 × g for 10 min at 4°C to remove dead cells, followed by centrifugation at 10,000 × g for 30 min at 4°C to eliminate cellular debris. The resulting supernatant was passed through a 0.22 µm filter and ultracentrifuged at 100,000 × g for 2 h at 4°C. The EV pellet was washed once with PBS and subjected to a second ultracentrifugation at 100,000 × g for 70 min. Finally, the purified EVs were resuspended in PBS containing trehalose, aliquoted, and stored at -80°C until further use. EV size distribution and concentration were determined by NTA.

**Quantitative real-time RT–PCR**

Total RNA was isolated from tissues or cells using Invitrogen TRIzol reagent (Thermo Fisher Scientific, Waltham, MA, USA). RNA purity (A260/A280) and integrity were evaluated by visualizing the 28S and 18S ribosomal RNA bands via agarose gel electrophoresis. Approximately 2 μg of total RNA was reverse transcribed into complementary DNA. The qRT-PCR was conducted using SYBR Green qPCR Master Mix (BioRad, Hercules, CA, USA) on a CFX 96 real-time PCR system with gene-specific primers (**Supplementary Table S2**). The thermal cycling conditions were: initial denaturation at 95°C for 10 min, followed by 40 cycles of 95°C for 15 s and 60°C for 1 min. Target gene expression levels were normalized using the 2^−ΔΔC^ method, with β-actin serving as the reference gene.

**Enzyme-Linked Immunosorbent Assay**

ELISA plates were coated with TM4SF1 antigen (10 μg/mL) overnight at 4°C. Non-specific binding sites were blocked using 3% bovine serum albumin (BSA) in PBS. Nanobody or EV solutions were serially diluted in 0.1% PBST (PBS with 0.1% Tween-20) and added to the coated plates. Following a 1-hour incubation at room temperature (RT), the plates were washed three times with 0.1% PBST. Detection was performed using an anti-HA HRP-conjugated antibody diluted in blocking buffer (3% BSA in 0.1% PBST, with a 1-hour incubation at RT. TMB substrate solution was added to each well and incubated at RT for 3–5 minutes until a blue color developed. The reaction was stopped by adding 50 μL of 1 M H₂SO₄ to each well. Absorbance was measured at 450 nm using a spectrophotometer.

**In-cell ELISA**

To evaluate the binding activity of nanobodies to cancer cell lines, in-cell ELISA was performed as follows: Cancer cell lines were seeded into 96-well plates at a density of 5 × 10⁴ cells per well and cultured overnight. Cells were fixed with 4% paraformaldehyde for 5 minutes, followed by blocking with 4% donkey serum solution at RT for 1 hour. Nanobody solutions were prepared by serial dilution (4000 nM to 62.5 nM) in 0.1% PBST and incubated with the cells for 1 hour at RT. After three washes with PBST, a rabbit anti-HA antibody (Creative Biomart, USA) was added and incubated at RT for 1 hour. The plates were washed again and then incubated with an Alexa Fluor 488-conjugated donkey anti-rabbit IgG antibody (Invitrogen, USA) for 1 hour at RT. Following a final set of three washes with PBST, fluorescence intensity was measured using the Sapphire Capture System (Sapphire, USA).

**Cellular uptake of EVs**

EVs at a concentration of 1 × 10¹¹ particles/mL were labeled with PKH67 dye (4 µL) and incubated at room temperature for 30 minutes in the dark. The labeled EVs were then centrifuged at 100,000 × g for 60 minutes at 4°C to pellet the vesicles. Tumor cells were seeded into 96-well plates at a density of 2 × 10⁴ cells per well and cultured for 24 hours. Labeled EVs were added to the tumor cells and incubated for 2 or 8 hours. After incubation, the cells were washed three times with PBS and fixed with 4% PFA solution for 30 minutes. Finally, cell nuclei were stained with DAPI solution, and the cells were visualized using a confocal microscope.

**Terminal deoxynucleotidyl transferase-mediated dUTP-biotin nick end labeling (TUNEL) staining**

Tissue sections were dewaxed and treated with DNase-free proteinase K (20 μg/mL; Beyotime Biotechnology, Hangzhou, Zhejiang, China) at 37°C for 30 minutes. Following three washes with PBS, 50 μL of TUNEL reaction mixture (Beyotime Biotechnology) was applied to the samples, which were incubated at 37°C for 60 minutes in the dark. The slides were then air-dried and sealed with anti-fade mounting medium. Fluorescence signals were observed using a confocal fluorescence microscope (Leica Biosystems, Wetzlar, Hessen, Germany).

**Immunofluorescence staining**

Tissue sections were fixed in a 4:1 acetone/methanol solution at −20°C for 10 minutes. To block non-specific binding, sections were incubated with 4% donkey serum in PBS for 30 minutes at RT. Primary antibodies were applied to the sections and incubated overnight at 4°C. The following day, sections were stained with fluorophore-conjugated secondary antibodies diluted in 0.5% donkey serum in PBS for 1 hour at RT in the dark. Finally, sections were mounted using a medium containing DAPI for nuclear staining.

**Immunohistochemical staining**

Tissue samples were fixed, embedded in paraffin, and sectioned for analysis. Paraffin-embedded sections were deparaffinized and rehydrated using xylene and graded ethanol solutions. Antigen retrieval was performed by steaming the slides in Tris-EDTA buffer (pH 9.0) for 15 minutes. To block endogenous peroxidase activity, the slides were incubated with 0.3% hydrogen peroxide (H₂O₂) in methanol (Aladdin, Shanghai, China) for 10 minutes. Non-specific binding was blocked by incubating the slides with 4% donkey serum in Tris-buffered saline containing 0.1% TBST for 1 hour at RT. Primary antibodies were applied to the slides and incubated overnight at 4°C. The following day, the sections were incubated with biotinylated secondary antibodies for 1 hour at RT, followed by 30 minutes with the VECTASTAIN Elite ABC Reagent (Vectorlabs, Burlingame, CA, USA). Staining was developed using 3,3′-diaminobenzidine (DAB; Vectorlabs) as the peroxidase substrate until the desired intensity was achieved. The slides were dehydrated through a graded ethanol-xylene substitute series, air-dried, and mounted with neutral mounting medium (Solaibao, Beijing, China).

**Western blotting analysis**

Total proteins were extracted using Radioimmunoprecipitation Assay (RIPA) buffer (Beyotime Biotechnology, Shanghai, China). Protein concentrations in the supernatants were determined using the BCA protein assay. Proteins were separated by sodium dodecyl sulfate-polyacrylamide gel electrophoresis (SDS-PAGE) using gels of 10%, 12.5%, or 15% polyacrylamide and subsequently transferred onto polyvinylidene fluoride (PVDF) membranes (Millipore, Billerica, MA, USA). Membranes were blocked with 5% nonfat dry milk (Biosharp, Hefei, Anhui, China) in Tris-buffered saline with 0.1% TBST for 1–2 hours at room temperature and incubated with primary antibodies overnight at 4°C. The membranes were then incubated with HRP-conjugated secondary antibodies. Protein bands were visualized using an enhanced chemiluminescence (ECL) detection reagent (GE Healthcare, Amersham, Buckinghamshire, UK).

**In vivo NIR-I fluorescence imaging**

H22 or Hepa1-6 tumor-bearing mice with a tumor volume of approximately 500 mm³ were randomly divided into two groups (n = 5). Mice were intravenously injected with DiR-labeled CAR.ctrl-EVs (1 × 10¹¹ particles) or CAR.A18-EVs (1 × 10¹¹ particles). Fluorescence imaging was performed at 4, 8, 12, and 24 hours post-injection using an in vivo near-infrared fluorescence imaging system (IVIS, PerkinElmer, USA). Mice were sacrificed 24 hours after injection, and tumors along with major organs (heart, liver, spleen, lungs, and kidneys) were harvested and imaged for fluorescence distribution.

**Pharmacokinetic Analysis Using ⁸⁹Zr-Based PET Imaging**

To evaluate the in vivo pharmacokinetic profile and biodistribution of CAR^TM4SF1^-EVs, purified CAR.A18-EVs and CAR.ctrl-EVs were labeled with ⁸⁹Zr using a DFO chelator. EVs were isolated from CAR^TM4SF1^-NKT cells via ultracentrifugation and further purified by immunoaffinity capture to ensure CAR⁺ EV enrichment. The radiolabeled EVs (1 × 10¹¹ particles) were intravenously injected into Hepa1-6 tumor–bearing C57BL/6 mice. PET imaging was performed at 2, 4, 8, 12, and 24 hours post-injection to assess real-time biodistribution in tumors, blood, and major organs (heart, liver, spleen, lungs, and kidneys). After the final scan, mice were euthanized, and the radioactivity of dissected tissues was quantified using γ-counting to calculate % ID/g values.

**Protein purification**

For the expression and purification of target proteins, recombinant plasmids pET-14B-TM4SF1 (human and mouse) were transformed into BL21(DE3) cells. Bacterial cultures were grown at 37°C with shaking at 225 rpm until reaching an optical density (OD600) of 0.6. Protein expression was induced with 0.2 mM IPTG at 16°C with shaking at 225 rpm overnight. Cells were harvested by centrifugation at 8000 × g for 15 minutes at 4°C, and the resulting pellets were resuspended in lysis buffer (300 mM NaCl, 50 mM NaH₂PO₄, 10 mM imidazole, pH 8.0, 1 mM PMSF). Lysis was performed via high-pressure homogenization on ice for three cycles. The lysate was clarified by centrifugation at 12,000 × g for 45 minutes at 4°C, and the supernatants were loaded onto a gravity column containing 1 mL of Ni-NTA agarose resin (Qiagen, Germany). The resin was washed sequentially with 50 mL Wash Buffer I (300 mM NaCl, 50 mM NaH₂PO₄, 20 mM imidazole, pH 8.0, 1 mM PMSF) and 50 mL Wash Buffer II (300 mM NaCl, 50 mM NaH₂PO₄, 40 mM imidazole, pH 8.0, 1 mM PMSF). Bound proteins were eluted with 25 mL Elution Buffer (300 mM NaCl, 50 mM NaH₂PO₄, 250 mM imidazole, pH 8.0, 1 mM PMSF). The eluted proteins were further purified by Superdex-150 gel filtration chromatography on an ÄKTA Pure System (GE Healthcare Life Sciences, USA) in 1× PBS. Purity was assessed by SDS-PAGE, and proteins were rapidly frozen in liquid nitrogen and stored at −80°C. Nanobodies were purified following the same Ni-NTA agarose resin protocol. Purified nanobodies were characterized by western blot analysis using anti-HA and anti-VHH antibodies.

**Flow analysis of immune cell infiltration in tumors**

Immune cell infiltration in tumor tissues from tumor-bearing mouse models was analyzed using a spectral flow cytometer (Cytek® Northern Lights™, Shanghai, China). Single-cell suspensions were prepared from collected tumor tissues by enzymatic digestion (RWD Life Science Co., Ltd, Shenzhen, China). Red blood cells were lysed using ACK buffer, and live cells were enumerated with Trypan Blue staining. Approximately 4 × 10⁶ cells per sample were resuspended in 100 μL FACS buffer. To block Fc receptors, cells were incubated with CD16/32 antibody at 4°C for 30 minutes. Fluorescently labeled antibodies specific to immune cell types were added, and samples were incubated on ice for 30 minutes. Macrophages were identified using surface markers CD45, F4/80, CD80, CD86 and CD206. Dendritic cells were detected with CD45, CD11c and MHC II, while T cells were characterized using CD45, CD3, CD4 and CD8 markers. Detection and analysis were performed using single-stained controls and sample tubes on a spectral flow cytometer (Cytek, USA).

**Detection of Memory T Cells in Spleen by Flow Cytometry**

Spleens were harvested from euthanized mice, and connective tissue was carefully removed. The spleens were cut into small pieces and dissociated into single-cell suspensions in FACS buffer (PBS with 1% BSA). The cell suspensions were filtered through a 70 μm filter membrane and centrifuged at 1000 × g for 5 minutes at 4°C. After discarding the supernatant, red blood cells were lysed using ACK buffer, followed by centrifugation at 500 × g for 10 minutes. The cell pellet was washed and resuspended in FACS buffer. To block Fc receptors, CD16/32 antibody was added and incubated briefly. Cells were then labeled with specific antibodies for memory T cell identification, including CD45, CD3, CD4, CD8, CD62L, and CD44.

**Transmission Electron Microscopy**

The morphology of EVs was analyzed using a TEM (JEOL JEM-1400 Plus, JEOL, Tokyo, Japan). Briefly, 30 μL of the EV sample was pipetted onto a carbon-coated copper grid (Xinxing Braim, Beijing, China) and allowed to adhere for 5 minutes. Excess liquid was gently removed, and the grid was stained with a drop of 4% uranyl acetate. After air-drying, the samples were imaged using TEM.

**Whole Transcriptome Sequencing**

Total RNA was extracted from frozen tissues using TRIzol reagent, following the manufacturer’s instructions. RNA purity was assessed using an ND1000 Nanodrop spectrophotometer, ensuring an A260:A280 ratio above 1.8 and an A260:A230 ratio above 2.0 for all samples. RNA integrity was evaluated with the Agilent 2200 TapeStation, and only samples with an RNA Integrity Number (RIN) above 7.0 were used for further processing. Ribosomal RNA was depleted using the Ribo-Zero™ Kit, and the remaining RNA was fragmented prior to first-strand and second-strand cDNA synthesis. The synthesized cDNA products were purified and enriched by PCR to generate the final cDNA libraries. Library quantification was performed using KAPA Library Quantification Kits according to the qPCR Quantification Protocol Guide, and library quality was verified with TapeStation D1000 ScreenTape. Indexed libraries were sequenced on an Illumina NovaSeq 6000 platform using paired-end reads.

***In vivo* subcutaneous model**

H22 and Hepa1-6 tumor cells were cultured under standard conditions and harvested using trypsin. A total of 1 × 10⁶ H22 or Hepa1-6 cells were injected subcutaneously into syngeneic Balb/c or C57BL/6 mice, respectively. Mice were euthanized if they exhibited significant signs of distress or when tumor size reached the ethical limit of 2000 mm³. Tumor volume was measured using calipers and calculated with the formula: (length × width²) / 2.

***In vivo* liver metastasis model**

Luciferase-expressing Hepa1-6 tumor cells were expanded under standard culture conditions and harvested using trypsin. To establish the liver metastasis model, anesthetized mice underwent splenic exposure, and tumor cells were injected into the spleens along their long axis. A total of 1 × 10⁶ Hepa1-6-Luc cells were slowly injected approximately 1 cm into the splenic tissue. Tumor growth was monitored via bioluminescence imaging (BLI) using the IVIS Spectrum in vivo imaging system (PerkinElmer) four minutes after an intraperitoneal injection of 3 mg D-luciferin (PerkinElmer). BLI values were quantified using Living Image software version 4.5 (PerkinElmer). Mice were randomized into treatment groups based on their BLI values, and treatments were initiated according to the study flowchart.

***In vivo* peritoneal metastasis model**

For the peritoneal metastasis model, 1 × 10⁶ Hepa1-6-Luc tumor cells were injected into the abdominal cavity. Mice were randomized and treated according to the procedures outlined for the orthotopic model.

***In vivo* lung metastasis model**

For the lung metastasis model, 1 × 10⁶ Hepa1-6-Luc tumor cells were injected into the tail vein. Mice were randomized and treated following the protocol established for the orthotopic model.

***In Vivo* Treatment Strategies**

**Specific treatment options are summarized in Supplementary Table 3**

To evaluate the therapeutic efficacy and safety of high or low doses of CAR.A18-NKT, CAR.ctrl-NKT and CAR.A18-EVs (Fig. 3A), H22 tumor-bearing mice were randomly assigned to seven treatment groups (n = 5): (1) Blank (PBS, 200 µL); (2) Low doses of CAR.A18-NKT (1 × 10^5^ cells/injection); (3) High doses of CAR.A18-NKT (1 × 10^6^ cells/injection); (4) Low doses of CAR.ctrl-NKT (1 × 10^5^ cells/injection); (5) High doses of CAR.ctrl-NKT (1 × 10^6^ cells/injection); (6) Low doses of CAR.A18-EVs (1 × 10^11^ particles/injection); (7) high doses of CAR.A18-EVs (1 × 10^12^ particles/injection) .

To assess the efficacy of CAR.A18-EVs in multiple tumor models, Hepa1-6 tumor-bearing mice were randomly divided into three groups (n = 5) and treated with: (1) Blank (PBS, 200 µL); (2) CAR.ctrl-EVs (1 × 10^12^ particles/injection); (3) CAR.A18-EVs (1 × 10^12^ particles/injection). Treatments were administered four times according to the schedule (Fig. 4A).

For the treatment of metastatic models, mice with hepatic, pulmonary, or abdominal metastases constructed from Hepa1-6 cells were randomly divided into two groups (n = 5) and treated four times with: (1) Blank (PBS, 200 µL); (2) CAR.A18-EVs (1 × 10^12^ particles/injection) (Fig. 4M-U).

To investigate the effects of macrophage or CD8^+^ T cell depletion on tumor growth, Hepa1-6 tumor-bearing mice in the pilot study were randomly assigned to six groups (n = 5) and treated with: (1) Blank (PBS, 200 µL); (2) Lyt3.2 (CD8b) antibody (i.p. 250 µg/mouse, every other day); (3) CLO (peritumoral injection daily, 1000 µg/mouse); (4) CAR.A18-EVs (1 × 10^12^ particles/injection) + Lyt3.2 antibody (i.p. 250 µg/mouse, every other day); (5) CAR.A18-EVs (1 × 10^12^ particles/injection); (6) CAR.A18-EVs (1 × 10^12^ particles/injection) + CLO (peritumoral injection daily, 1000 µg/mouse). Treatments were administered according to the schedule (Fig. 5I).

To evaluate the relative contributions of OXPHOS inhibition and ER stress activation to the antitumor effects of CAR.A18-EVs, Hepa1-6 subcutaneous tumor–bearing mice were randomly assigned to six groups (n = 5) and treated as follows (Fig. S14A): (1) Blank (PBS, 200 µL); (2) CAR.ctrl-EVs (1 × 10¹² particles/injection); (3) CAR.A18-EVs (1 × 10¹² particles/injection); (4) Rotenone (i.p. 2 mg/kg/day)); (5) CAR.A18-EVs (1 × 10¹² particles/injection) + DCA (i.p. 50 mg/kg/day)); (6) CAR.A18-EVs (1 × 10¹² particles/injection) + 4-PBA (p.o. 1 g/kg/day). Tumor volume, individual tumor growth curves, and survival were monitored throughout the experiment.

For combination immunotherapy, Hepa1-6 tumor-bearing mice were randomly divided into four groups (n = 5): (1) Blank (PBS, 200 µL); (2) PD-1/CTLA-4 antibody (i.v. 10 mg/kg); (3) CAR.A18-EVs (1 × 10^12^ particles/injection); (4) CAR.A18-EVs (1 × 10^12^ particles/injection) + PD-1/CTLA-4 antibody (i.v. 10 mg/kg). Treatments were administered according to the schedule (Fig. 6A, F).

Statistical Analysis

No data pre-processing (including data transformation, normalization, or outlier removal) was performed prior to statistical testing. Unless otherwise specified, data are presented as the mean ± SD. Sample sizes (n) are provided in each figure legend. Differences between two samples were analyzed using an unpaired two-tailed Student’s t-test. One-way ANOVA was employed to compare tumor weights and various toxicological parameters among four groups, while two-way ANOVA was used to assess in vitro cell viability and tumor growth curves. Survival curves were compared using log-rank tests. A p-value of less than 0.05 was considered statistically significant. Asterisks indicate significant differences (*p < 0.05, **p < 0.01, ***p < 0.001). Statistical analyses were performed using GraphPad Prism 10 software.

**Table S1. The primary** **antibody used in this study.**

| Antibody | Catalogue number | Supplier name |
| --- | --- | --- |
| CD9 | ab236630 | Abcam |
| Alix | ab275377 | Abcam |
| CD63 | ab134045 | Abcam |
| CD81 | ab79559 | Abcam |
| TSG101 | ab125011 | Abcam |
| Calnexin | ab133615 | Abcam |
| Calreticulin | ab92341 | Abcam |
| ERp57 | ab10287 | Abcam |
| ATF4 | ab85049 | Abcam |
| GRP94 | ab3674 | Abcam |
| FasL | ab302905 | Abcam |
| CHOP | 15204-1-AP | proteintech |
| Camelid VHH Antibody | A01861 | GenScript |
| Camelid VHH Antibody [iFluor 647] | A02019 | GenScript |
| Alexa Fluor® 488 anti-HA-Tag antibody | 901509 | BioLegend |
| HA-tag antibody | M20003 | Abmart |
| P65 antibody | A19653 | ABclonal |
| Phospho-P65 antibody | AP1294 | ABclonal |
| FITC anti-mouse CD3 Antibody | 100203 | Biolegend |
| PE anti-mouse CD45 | 147711 | Biolegend |
| F4/80 | MCA497R | Bio-RAD |
| PERCP-CY5.5-MHC II Antibody | #16114 | Cell Signaling |
| APC anti-mouse CD4 Antibody | 100411 | Biolegend |
| APC/Cyanine7 anti-mouse CD86 Antibody | 159217 | Biolegend |
| PE anti-mouse CD11C Antibody | 117307 | Biolegend |
| PE anti-mouse CD8b.2 Antibody | 140408 | Biolegend |
| Brilliant Violet 421™ anti-mouse CD62L Antibody | 104435 | Biolegend |
| Alexa Fluor® 700 anti-mouse CD44 Antibody | 156009 | Biolegend |
| Anti-mouse CD8β (Lyt 3.2) Antibody | A2137 | Selleckchem |
| Anti-mouse PD-1 (CD279) Antibody | A2122 | Selleckchem |
| Anti-mouse CTLA-4 (CD152) Antibody | A2103 | Selleckchem |

**Table S2. List of genes examined by real-time PCR and the primer sequences used.**

| Gene name | Forward primer | Reverse primer |
| --- | --- | --- |
| *m_IFNB1* | *GCCTTTGCCATCCAAGAGATGC* | *ACACTGTCTGCTGGTGGAGTTC* |
| *m_TNF* | *GGTGCCTATGTCTCAGCCTCTT* | *GCCATAGAACTGATGAGAGGGAG* |
| *m_Ccl2* | *GCTACAAGAGGATCACCAGCAG* | *GTCTGGACCCATTCCTTCTTGG* |
| *m_Ccl3* | *ACTGCCTGCTGCTTCTCCTACA* | *ACTGCCTGCTGCTTCTCCTACA* |
| *m_Ccl4* | *ACCCTCCCACTTCCTGCTGTTT* | *CTGTCTGCCTCTTTTGGTCAGG* |
| *m_Ccl5* | *CCTGCTGCTTTGCCTACCTCTC* | *ACACACTTGGCGGTTCCTTCGA* |
| *m_Ccl20* | *GTGGGTTTCACAAGACAGATGGC* | *CCAGTTCTGCTTTGGATCAGCG* |
| *m_Cxcl1* | *TCCAGAGCTTGAAGGTGTTGCC* | *AACCAAGGGAGCTTCAGGGTCA* |
| *m_Cxcl2* | *CATCCAGAGCTTGAGTGTGACG* | *GGCTTCAGGGTCAAGGCAAACT* |
| *m_Cxcl10* | *ATCATCCCTGCGAGCCTATCCT* | *GACCTTTTTTGGCTAAACGCTTTC* |
| *m_Il12* | *GCGGCATGTTCTGGATTTGACT* | *CCACCACAGTTGCTGACTCAT* |
| *m_Il6* | *TACCACTTCACAAGTCGGAGGC* | *CTGCAAGTGCATCATCGTTGTTC* |
| *m_Il1b* | *TGGACCTTCCAGGATGAGGACA* | *GTTCATCTCGGAGCCTGTAGTG* |
| *m_β-actin* | *GGCTGTATTCCCCTCCATCG* | *CCAGTTGGTAACAATGCCATGT* |

**Table S3. Drug doses and treatment frequencies for different experimental schemes.**

| **Therapy regimen** | **Treatment Frequency** | **EV dose (per injection)** | **Lyt3.2 antibody dose** | **CLO dose** | **Rotenone dose** | **DCA dose** | **4-PBA dose** | **PD-1 antibody dose** | **CTLA-4antibody dose** | **Figures** |
| --- | --- | --- | --- | --- | --- | --- | --- | --- | --- | --- |
| CAR.ctrl-EVs | 4×CAR.ctrl-EVs | 1×10^12^ particles |  |  |  |  |  |  |  | 4A-L, S14 |
| CAR.A18-EVs | 4×CAR.A18-EVs | 1×10^12^ particles |  |  |  |  |  |  |  | 4, 5, 6, S14 |
| CAR.A18-EVs + Lyt3.2 | 4×CAR.A18-EVs + 7×Lyt3.2 | 1×10^12^ particles | 250 µg |  |  |  |  |  |  | 5I-K |
| CAR.A18-EVs + CLO | 4×CAR.A18-EVs + 7×CLO | 1×10^12^ particles |  | 1000 µg |  |  |  |  |  | 5I-K |
| Rotenone | 6× Rotenone |  |  |  | 2 mg/kg/day |  |  |  |  | S14 |
| CAR.A18-EVs + DCA | 4×CAR.A18-EVs +  6×DCA | 1×10^12^ particles |  |  |  | 50 mg/kg/day |  |  |  | S14 |
| CAR.A18-EVs + 4-PBA | 4×CAR.A18-EVs +  7×4-PBA | 1×10^12^ particles |  |  |  |  | 1 g/kg/day |  |  | S14 |
| CAR.A18-EVs + PD-1 | 2×CAR.A18-EVs + 2×PD-1 | 1×10^12^ particles |  |  |  |  |  | 10 mg/kg |  | 6A-E |
| CAR.A18-EVs + CTLA-4 | 2×CAR.A18-EVs + 2×CTLA-4 | 1×10^12^ particles |  |  |  |  |  |  | 10 mg/kg | 6F-J |

**References**

[1] H. Du, K. Hirabayashi, S. Ahn, N. P. Kren, S. A. Montgomery, X. Wang, K. Tiruthani, B. Mirlekar, D. Michaud, K. Greene, S. G. Herrera, Y. Xu, C. Sun, Y. Chen, X. Ma, C. R. Ferrone, Y. Pylayeva-Gupta, J. J. Yeh, R. Liu, B. Savoldo, S. Ferrone, G. Dotti, "Antitumor Responses in the Absence of Toxicity in Solid Tumors by Targeting B7-H3 via Chimeric Antigen Receptor T Cells," *Cancer Cell* **2019**, *35* (2), 221, <https://doi.org/10.1016/j.ccell.2019.01.002>.

[2] X. Zhou, Y. Wang, Z. Dou, G. Delfanti, O. Tsahouridis, C. M. Pellegry, M. Zingarelli, G. Atassi, M. G. Woodcock, G. Casorati, P. Dellabona, W. Y. Kim, L. Guo, B. Savoldo, A. Tsagaratou, J. J. Milner, L. S. Metelitsa, G. Dotti, "CAR-redirected natural killer T cells demonstrate superior antitumor activity to CAR-T cells through multimodal CD1d-dependent mechanisms," *Nat Cancer* **2024**, *5* (11), 1607, <https://doi.org/10.1038/s43018-024-00830-0>.

[3] K. Hirabayashi, H. Du, Y. Xu, P. Shou, X. Zhou, G. Fuca, E. Landoni, C. Sun, Y. Chen, B. Savoldo, G. Dotti, "Dual Targeting CAR-T Cells with Optimal Costimulation and Metabolic Fitness enhance Antitumor Activity and Prevent Escape in Solid Tumors," *Nat Cancer* **2021**, *2* (9), 904, <https://doi.org/10.1038/s43018-021-00244-2>.
